# Safety assessment of a proprietary fermented soybean solution, Symbiota®, as an ingredient for use in foods and dietary supplements: Non‐clinical studies and a randomized trial

**DOI:** 10.1002/fsn3.3921

**Published:** 2024-01-08

**Authors:** Chien‐Min Hung, Wen‐Cheng Chu, Wen‐Yen Huang, Pei‐Jung Lee, Wen‐Chih Kuo, Cheng‐Yu Hou, Chia‐Chun Yang, Ai‐Jen Yang, Wei‐Kai Wu, Ming‐Liang Kuo, Ming‐Shiang Wu, Wan‐Jiun Chen

**Affiliations:** ^1^ Microbio Co., Ltd Taipei Taiwan; ^2^ Microbio (Shanghai) Biotech Company Shanghai China; ^3^ Department of Medical Research National Taiwan University Hospital Taipei Taiwan; ^4^ Department of Internal Medicine National Taiwan University Hospital Taipei Taiwan; ^5^ Department of Internal Medicine National Taiwan University College of Medicine Taipei Taiwan

**Keywords:** food safety, metabolites, probiotics, symbiotic fermentation

## Abstract

A safety evaluation was performed of Symbiota®, which is made by a proprietary anaerobic fermentation process of soybean with multistrains of probiotics and a yeast. The battery of genotoxicity studies showed that Symbiota® has no genotoxic effects. Safety and tolerability were further assessed by acute or repeated dose 28‐ and 90‐day rodent studies, and no alterations in clinical observations, ophthalmological examination, blood chemistry, urinalysis, or hematology were observed between the control group and the different dosing groups (1.5, 5, and 15 mL/kg/day). There were no adverse effects on specific tissues or organs in terms of weight and histopathology. Importantly, the Symbiota® treatment did not perturb hormones and other endocrine‐related endpoints. Of note, the No‐Observed‐Adverse‐Effect‐Level was determined to be 15 mL/kg/day in rats. Moreover, a randomized, double‐blind, placebo‐controlled clinical trial was recently conducted with healthy volunteers who consumed 8 mL/day of placebo or Symbiota® for 8 weeks. Only mild adverse events were reported in both groups, and the blood chemistry and blood cell profiles were also similar between the two groups. In summary, this study concluded that the oral consumption of Symbiota® at 8 mL/day by the general population does not pose any human health concerns.

## Graphical abstract

This study provided a comprehensive safety assessment through the battery of guideline‐compliant pre‐clinical studies and a placebo‐controlled clinical trial to support the safety of Symbiota® (MS‐20) consumption as a nutritional and functional ingredient in selected foods for the general population within the proposed use levels. Image created with BioRender.com.

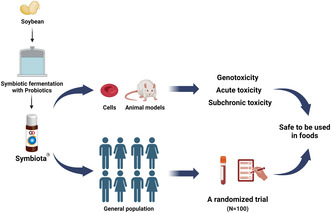

## 1 INTRODUCTION

Symbiota® is a next‐generation fermented soybean solution meticulously made through expert anaerobic fermentation using a unique combination of bacterial strains, including *Lacticaseibacillus paracasei*, *Lactobacillus delbrueckii* subsp. *bulgaricus*, *Bifidobacterium longum*, along with a singular yeast strain, *Saccharomyces cerevisiae*. The final product of Symbiota® does not contain live microorganisms, as they are heat‐inactivated and removed by filtration. Therefore, the final product of Symbiota® is mainly composed of water, organic acids derived from fermentation (e.g., lactic acid and acetic acid), proteins, lipids, carbohydrates, amino acids, minerals, isoflavones, and other microbial metabolites. Symbiota® is intended for use as a nutritional or functional ingredient in selected foods for the general population.

Fermented soybean products (FSP) have been extensively studied and diverse biological functions were reported. A fermented soybean product was shown to constrain the growth of human breast cancer MCF‐7 cells by eliciting reactive oxygen species generation (Chang et al., 2002). Conversely, another study showed that FSP can act as an antioxidant to eradicate free radicals in vitro, and it can enhance the activities of antioxidant‐associated enzymes, such as catalase, superoxide dismutase, and glutathione peroxidase, in the liver when FSP was orally administrated (Hu et al., 2004). The findings suggest the distinct functions of FSP in protecting normal cells from genotoxic damage while inducing cell death in abnormal cells such as tumor cells. In addition, a study by Chin et al. demonstrated that FSP has antibacterial activity against various bacteria, including oxacillin‐resistant *Staphylococcus aureus* (ORSA) and vancomycin‐resistant *Enterococcus faecalis* (VRE; Chin et al., 2012). The researchers also observed that the mice fed with FSP exhibited enhanced proliferation of T helper (Th1/Th2) cells and elevated activity of natural killer (NK) cells (Chin et al., 2014). This observation was further supported by the clinical trial conducted by Tsang et al., which showed a significant preservation of NK cell activities in the patients receiving both FSP and chemotherapy compared to those who received chemotherapy alone (Tsang et al., 2007). Remarkably, the clinical study conducted by Chi et al. demonstrated that when Symbiota®, also known as MicrSoy‐20 (MS‐20), was used as a chemotherapeutic adjuvant, the MS‐20 treatment group experienced significant alleviation of chemotherapy‐induced adverse effects, such as fatigue and appetite loss, while quality of life (QoL) was improved (Chi et al., 2014). These results underscore the potential of Symbiota® (MS‐20) in advancing disease treatment and enhancing the well‐being of patients.

Although previous studies have helped understand the diverse functions of FSP, a comprehensive evaluation of the safety profile is warranted since Symbiota® is proposed to be consumed by the general population. Therefore, this work aims to determine the safety and tolerability of Symbiota® through a series of toxicology studies in model cell lines and animals. To assess mutagenic effects and genotoxic hazards, in vitro gene mutation and chromosome aberration tests in Chinese hamster ovary cells, and an in vivo erythrocyte micronucleus test in mice were performed. Subsequently, acute or repeated dose 28‐ and 90‐day oral toxicity studies in Sprague–Dawley rats were performed to further assess potential systemic toxic effects. This study further reported the results of a double‐blind, randomized, placebo‐controlled trial for the assessment of safety in humans. This clinical trial (registered at ClinicalTrials.gov as NCT04674839) was conducted with healthy participants and assessed the following endpoints: vital signs, physical examinations, clinical laboratory tests, treatment emergent adverse events (TEAEs), and serious adverse events (SAEs). The impact on the gut microbiome of the subjects was also assessed but these data are not reported herein.

## 2 MATERIALS AND METHODS

### 2.1 Test product

Symbiota® (MS‐20) fermented soybean solution (Microbio Co., Ltd., Taipei, Taiwan) is manufactured in a cGMP facility certified with HACCP, ISO 9001, ISO 22000, ISO 14001, and ISO/IEC 17025 quality systems. All fermentation microorganisms were acquired from the Bioresource Collection and Research Center (BCRC), Taiwan, and did not receive any genetic manipulation. The manufacturing started with the anaerobic fermentation of non‐GMO soybean (*Glycine max* (*L*.) *Merr*.) with multi‐strain probiotic bacteria, such as *Lacticaseibacillus paracasei* (BCRC 12188), *Lactobacillus delbrueckii* subsp. *bulgaricus* (BCRC 10696), *Bifidobacterium longum* (BCRC 80229), and a yeast strain, *Saccharomyces cerevisiae* (BCRC 20262). The final steps involved the heat inactivation of fermentation microorganisms (95°C for 30 min) and the filtration with a 0.22‐μm ceramic filter. The final product weighs 1.136 g/mL with a pH of 4.0 ± 0.2. The composition of Symbiota® is summarized in Table 1, and, notably, the isoflavone content of the ingredient is compared to a Generally Recognized as Safe (GRAS) ingredient and other soy‐derived foods (Table 2). The suggested daily intake levels are 1–8 mL/day for the general population as a food ingredient or as a dietary supplement.

**TABLE 1:** The composition of Symbiota®.

Component	Percentage (w/w)
Moisture	68.96
Crude protein	9.13
Crude fat	0.66
Ash	4.31
Carbohydrate^a^	16.94
*Lactic acid* ^b^	8.65
*Acetic acid* ^b^	0.29
*Geinstein* ^b^	0.005
*Daidzein* ^b^	0.012
*Glycitein* ^b^	0.002

^a^ %Carbohydrate = 100 − %Moisture − %Crude protein − %Crude fat − %Ash (21 CFR 101.9).

^b^ Quantified using high‐performance liquid chromatography (HPLC).

**TABLE 2:** Total isoflavone content of Symbiota® and other soy‐derived products.

	Symbiota®	Soy yogurt (NDB no. 99510)^a^	Soy protein drink (NDB no. 99560)^a^	Soy protein hydrolysate (GRN #134)^b^
Genistein	5.0 ± 1.5	13.77	42.91	96
Daidzein	10.5 ± 3.0	16.59	27.98	49
Glycitein	2.5 ± 0.8	2.80	10.76	9.5
Total isoflavones	18 ± 5.3	33.17	81.65	154.5

*Note*: Unit: mg/100 g.

^a^ USDA Database for the Isoflavone Content of Selected Foods, Release 2.1 (November 2015).

^b^ https://www.fda.gov/food/generally‐recognized‐safe‐gras/gras‐notice‐inventory.

The stability assessment of Symbiota® was conducted under controlled conditions. Three batches of Symbiota (B607293082, B608193087, and B610122001) were subjected to testing at 0, 1, 3, 6, 9, 12, 18, 24, 30, 36 months for the normal storage condition (25°C with 60% RH). Other accelerated or exaggerated conditions (30°C with 75% RH, 35°C with 75% RH, and 40°C with 75% RH) were conducted and tested only at 0, 1, 3, and 6 months. Each sample was retrieved from its original package for the indicated time points and was analyzed for the properties of appearance, specific gravity, pH value, composition, and microorganisms. The study has been conducted for 3 years, and the index components (Lactic acid and Daidzein) and other properties remain within the specifications, implying Symbiota® is stable under normal storage conditions. All microbiology parameters (aerobic plate count, total yeasts and molds, *E. coli*, *S. aureus*, and *Salmonella*) were tested negative at each time point.

### 2.2 Animals

ICR mice and Crl:CD (standard deviation (SD)) rats used in the studies conducted by the Development Center of Biotechnology (DCB), Taiwan, were obtained from the National Laboratory Animal Breeding and Research Center, Taipei, Taiwan. Animals were quarantined and housed in an AAALAC‐accredited facility which was maintained at 21 ± 2°C and 50 ± 20% relative humidity with a 12‐h light/12‐h dark cycle. Healthy mice of the same sex were randomly assigned to groups of up to five per cage in polycarbonate cages. The rats were housed two per cage in stainless‐steel wire mesh cages. In the 90‐day subchronic toxicology study, the SD rats were purchased from BioLASCO Taiwan CO., Ltd, and were housed in a controlled room of 22 ± 3°C and 50 ± 20% relative humidity with a 12‐h light/12‐h dark cycle. These animals were kept in autoclaved polyethylene cages with two rats per cage for the main study group and one rat per cage for the recovery study group. In general, animals were acclimated for 6–7 days before dose administration and were randomized into groups with weight variation not exceeding ±20% of the mean body weight. All animals had free access to rodent diet 5010 and to drinking water, except the feed was removed during the fasting period. The protocols were approved by the Institutional Animal Care and Use Committee (IACUC).

### 2.3 In vitro gene mutation test

A GLP‐compliant (OECD, 1997a) in vitro mammalian cell gene mutation test using the Hprt gene was conducted at DCB, Taiwan. The assays were performed based on OECD Guideline TG 476 (OECD, 1997d). The CHO‐Kl cells (ATCC CCL‐61) were purchased from the American Type Culture Collection. After thawing, cells were maintained in McCoy's 5A medium (GIBCO) with 10% fetal bovine serum and NaHCO₃ (pH 7.0~7.2). The S9 preparation was obtained from MOLTOX.

In the dose‐finding experiment, 100 and 50 μL/mL Symbiota® (MS‐20) were set as the top concentrations for the gene mutation assay without or with Aroclor 1254‐induced rat liver S9, respectively. Methanesulfonic acid, ethyl ester (EMS) (Sigma M0880) at 0.1 μL/mL, and *N*‐nitroso‐dimethylamine (DMN; Sigma N7756) at 100 μg/mL were utilized as positive controls for the tests in the nonactivated or S9‐activated conditions, respectively. CHO‐Kl cells at the exponential growth phase were seeded in complete McCoy's 5A medium at 1 × 10^6^ cells per 100‐mm dish overnight. Each duplicate dish was incubated with the indicated treatment at 37°C for 5 h. The cells were washed with PBS and then were supplemented with a fresh medium for another 18~24 h. For the estimation of cytotoxicity, each treated dish was cultured independently at approximately 200~1600 cells in 60‐mm dishes for 7 days. For mutant expression, the replicates from each condition were cultured independently every 2–3 days during the 7‐day expression period. For the selection of the 6‐thioguanine (6‐TG)‐resistant phenotype, cells from each condition were plated into five dishes at a density of 2 × 10^5^ cells/100‐mm dish with 10 μM 6‐TG. For cloning efficiency, 200–400 cells per 60‐mm dish were seeded in triplicate in the absence of 6‐TG. After 7 days of incubation, the colonies were fixed, stained, and counted for both mutant frequency and cloning efficiency. The mutation frequency was calculated as the number of mutant colonies divided by the number of cells selected, which was then normalized by the cloning efficiency of each treatment and shown as mutants per 10^6^ clonable cells.

A positive response for the test article was determined when it fulfilled both criteria: (1) two consecutive concentrations are showing mutant frequencies of ≧40 mutants per 10^6^ clonable cells, and (2) There is a dose–response increase in mutant frequencies.

### 2.4 In vitro chromosomal aberration test

In accordance with OECD Guideline 473 (OECD, 1997b), the assays were performed at a GLP‐compliant laboratory (OECD, 1997a), DCB, Taiwan. The target cell line and reagents were the same as described above (Section 2.2). CHO‐K1 cells were initially plated at 5 × 10^5^ cells/60‐mm dish and cultured overnight before the treatments. Chromosome aberration assays were performed with three schemes. Scheme I, cells were incubated with the test article for 3 h in the absence of S9. Scheme II, cells were treated for 3 h with S9 activation. In these two test schemes, the cells were kept growing in a fresh medium, and mitotic cells were harvested at about 20 h from the initiation of treatment. In Scheme III, the cells were treated for a prolonged period (20 h) in the medium without S9. Symbiota® was tested at 6.25, 12.5, 25, 50, and 100 μL/mL. Mitomycin C (Boehringer Mannheim Art.107409) at 1 μM for a 3‐h treatment was used as the positive control. Cyclophosphamide (Sigma #C0768) at 40 μM for a 3‐h treatment was the positive control for the scheme with S9 activation. 0.1 μg/mL Colcemid was included in the medium during the last 2 h of incubation before harvest. Mitotic cells were collected and fixed by a mixture of acetic acid and methanol (1:3). The frequency of aberrant cells in the concurrent negative control must not exceed 3%. The percentage of aberrant cells in the concurrent positive controls should be statistically increased (*p* < .05) relative to the negative control. For a given treatment, the percent of aberrant cells is analyzed by a one‐tailed binomial test and compared to the negative control individually. The significant level was set as *p*‐value <.01 based on a standard normal distribution.

### 2.5 Mammalian erythrocyte micronucleus test

A micronucleus assay was conducted following OECD Guideline 474 (OECD, 1997c) with GLP‐compliance (OECD, 1997a) at DCB, Taiwan. 8–9‐week‐old ICR mice were employed. Three males/females per dose group were used in the preliminary range‐finding study; five males/dose group were used in the definitive micronucleus assay. Injection water was used to prepare the test product solution. Animals were fasted for approximately 4 h before dosing, and the test product solution was administered to mice by gavage once daily for two consecutive days. Since there were neither mortality nor significant toxicity signs in the observation period at a dose of 20 mL/kg in a preliminary range‐finding analysis, the highest test dose was set as 20 mL/kg MS‐20 (Group 4). Lower doses of 10 and 5 mL/kg MS‐20 were also tested (Group 3 and Group 2). Mitomycin C (1.0 mg/kg) was adopted as a positive control (Group 5). Vehicle‐treated animals were also included as a negative control (Group 1).

Micronuclei (MN) incidence was examined in reticulocytes at 36–48 h after the last treatment administration. The positive control (1 mg/kg mitomycin C) was dosed i.p. once, and blood was collected approximately 48 h after dosing. A minimum of two thousand reticulocytes were measured for the incidence of micronuclei for each animal. The data are represented as the number of MN per 1000 RETs (MN/1000 RETs), and the mean ± SD of each group was calculated. The proportion of reticulocytes to total erythrocytes (RET ratio) was recorded for each animal as an indicator of any toxicity of bone marrow associated with treatment. A positive response was defined as if a dose‐dependent escalation in micronucleated reticulocytes was observed and the frequency for at least one dose was statistically increased compared to the vehicle control (*p* < .05, *T*‐test). All assay criteria for a valid experiment were met according to OECD 474.

### 2.6 Acute oral toxicity study

The study was performed at the GLP‐compliant laboratory (OECD, 1997a), DCB, Taiwan. Six‐week‐old Crl:CD (SD) rats with body weight ranges of 172–191 g for males and 133–147 g for females. The protocol was approved by the IACUC and followed OECD Guideline 401 (OECD, 1995). A total of 24 males and 24 females were divided into four groups, and each group consisted of six males and six females. The control group was orally administered with injection‐grade water. The remaining groups were dosed with MS‐20 at low (5 mL/kg), middle (10 mL/kg), or high (20 mL/kg) doses, respectively, by oral gavage. Half of each dosing volume was administrated separately with a 2‐h interval. Mortality and clinical signs were monitored at 1, 2, 3, and 4 h after the second dosing. In the following days, the animals were examined twice daily. Rats were weighed prior to dosing (Day 1), weekly thereafter (Day 8), and before necropsy (Day 14). Gross necropsies were performed on all rats that survived at the end of the study (Day 14). The survivors were euthanized with an overdose of sodium pentobarbital solution by i.p. injection and necropsied in a randomized order.

### 2.7 Repeated dose 28‐day oral toxicity study in rats

The study was performed by DCB as previously described. Eighty SPF SD rats (40 females and 40 males), 6‐week‐old at the initiation of the study with body weights from 198 to 252 g for males and from 157 to 192 g for females, were randomized into four groups (10 females and 10 males per group). The dose levels were 0 (vehicle control), 1.5 (low), 5 (middle), and 15 (high) mL/kg.

The experimental treatment solutions were prepared with injection‐grade water at three dose levels (1.5, 5, and 15 mL/kg) and were administered to rats (10 males/females for each dosing group) once every morning by gavage for 28 days. The dosing volume was determined according to the most recently measured body weight for each animal, and 1–10‐ml plastic disposable syringes (needle size: 16‐gauge, 80 mm in length) were used. The rats were monitored for clinical signs and mortality after dosing. Body weights were measured on all rats before the first dosing day (Study Day 1, SD1), weekly thereafter for the duration of the study period, before the termination of the study (SD28), and on the day of necropsy (SD29). The food consumption for two rats in each cage was measured weekly throughout the study period. Gross examination of the eyes was performed on all four groups of animals on the days before the first dosing and prior to the termination of the study on SD28. Urine specimens were individually collected for 16 h on SD28 for urinalysis. All rats were fasted for over 16 h before necropsy (SD29). Plasma samples were obtained through the femoral vein with sampling tubes containing EDTA for hematology and clinical chemistry. Organs, including the brain, liver, kidneys, heart, adrenals, thymus, spleen, and testes (males only), were taken from all surviving animals at the end of the study (SD29). Representative samples of the tissues collected at necropsy were fixed in 10% buffered formalin or a 3% glutaraldehyde solution for histopathology. The study processes were inspected for compliance with Good Laboratory Practices (GLP) and the study protocol was designed in line with the OECD 407 guideline (OECD, 1981).

### 2.8 Repeated dose 90‐day subchronic oral toxicity study in rats

The 90‐day subchronic study was conducted at the MedGaea Life Sciences Institute (Taipei, Taiwan). The study complied with the principles of Good Laboratory Practice for Nonclinical Laboratory Studies (FDA, 2022; OECD, 1997a). The protocol was following the OECD Guidelines TG 408 (OECD, 2018). To inspect the possible systemic toxicity of Symbiota® in SD rats, male and female rats at the age of 6 weeks were divided into four groups (control, low dose, middle dose, and high dose), each consisting of ten male and ten female rats. An additional five animals per sex for both the control (Re‐C) and high‐dose (Re‐H) groups were allowed 14 days of recovery after the 90‐day treatment to demonstrate the reversibility, persistence, or latency of toxic effects.

The Symbiota® solution (lot no. FB4011) was delivered to the rats via oral gavage at 1.5 mL/kg/day (Symbiota® low‐dose group, S‐L), 5.0 mL/kg/day (Symbiota® middle‐dose group, S‐M) and 15.0 mL/kg/day (Symbiota® high‐dose group, S‐H) for 90 consecutive days, and sterile water was administrated as vehicle control. During the period of the study, all animals were monitored for general health conditions and clinical signs of illness or discomfort twice daily for up to 13 weeks for the main study and 15 weeks for the recovery study. Detailed clinical observations were performed 1 day before administration and once a week thereafter in all animals. All rats were sacrificed at the end of the observation period for further toxicological examinations.

Each animal was weighed on Day 1 prior to administration, once every week during the study period, and before sacrifice. Food consumption was assessed once a week by the difference between the known amount of feed on a weighing day and the amount left 1 week later (g/one or two rat(s)/cage). Ophthalmological examination was performed the day prior to administration and before sacrifice. Urine samples were collected for urine chemistry and sediment analysis. Hematology measurements were conducted at the end of the study. The prothrombin time (PT) and activated partial thromboplastin time (APTT) were examined using a blood coagulated analyzer (CA‐500, Sysmex, Japan). Blood was collected after fasting for at least 8 h and centrifuged for 15 min to collect serum. Serum samples were then analyzed by an automated analyzer for biochemistry (HumaStar 100, Human, Germany). Thyroid hormones were analyzed for total T4 (Cusabio Technology, LLC, Lot no. C2230042287), T3 (Cusabio Technology LLC, Lot no. C2230022285), and TSH (MyBioSource Inc., Lot no. NM04 T6BF3926). All rats were euthanized by CO_2_ inhalation for necropsy. All organs were examined and weighed, and histopathology and gross findings were recorded.

### 2.9 Human clinical safety trial in healthy subjects

**FIGURE 1:**
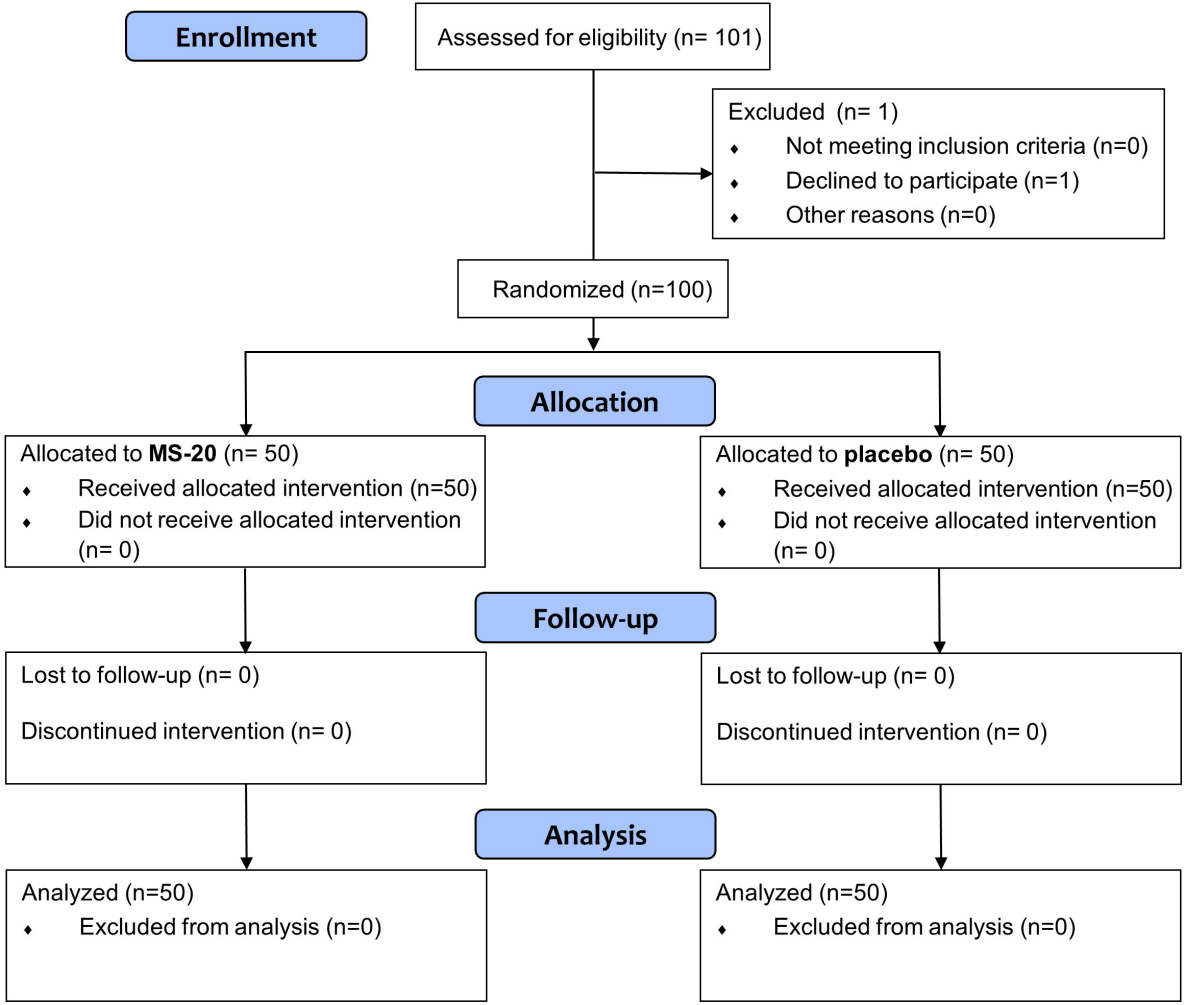
Flow diagram for the clinical study. A total of 101 subjects who met the criteria of this study signed the written informed consent, but one withdrew the consent. Eligible subjects were randomly assigned to receive either placebo or Symbiota® (MS‐20) in a ratio of 1:1. The data of 50 participants per group were analyzed.

A double‐blind, randomized, and placebo‐controlled trial for the assessment of the impact of Symbiota® (MS‐20) in healthy people was conducted by the National Taiwan University Hospital (NTUH) (Study Number: 201903096MIPB) and was registered at ClinicalTrials.gov as NCT04674839. The trial was carried out in compliance with applicable standard operating procedures and recognized international standards, including GLP, ICH Good Clinical Practices (GCP), and the *Declaration of Helsinki* (revised in 2013). From October 18, 2019 to November 20, 2020, a total of 101 subjects who met the criteria of this study signed the written informed consent, but one withdrew the consent (Figure 1). These subjects were randomly assigned to receive either placebo or Symbiota® (MS‐20) in a ratio of 1:1 with 50 participants per group. The sample size was designed to have a power of at least 80% for a two‐sided test at the 0.05 level of significance. The computer‐generated block randomization scheme was prepared by an independent qualified biostatistician. Investigators and research staff remained blinded to the randomization list until the database was locked. Only the authorized personnel was informed of the randomized treatment assignment in a sealed envelope containing the individual treatment code at the baseline visit. The eligibility criteria were as follows: male or female volunteers aged between 20 and 65 years old, subjects who agreed to comply with all requirements of the study protocol, and participants not taking any prebiotic and probiotic supplements during the trial. Additionally, all medications used by subjects were documented, except vancomycin and fluoroquinolones were prohibited for the entire trial. The complete trial protocol can be found at ClinicalTrials.gov.

The study protocol included a 7‐day screening period, an 8‐week intervention period, and an 8‐week follow‐up period. 4 mL MS‐20 or placebo was orally consumed by the subjects twice per day in the morning and the afternoon for the treatment period. The placebo solution is similar in appearance to MS‐20 but contains no microbial metabolites. The primary endpoint of this clinical trial was to assess the changes in gut microbiota, which are subject to further investigation in another study. Safety‐related measurements including vital signs (heart rate, respiratory rate, oral body temperature, systolic and diastolic blood pressures), physical examinations, clinical laboratory test results (homocysteine, fasting blood sugar, high sensitive C‐reactive protein, AST, creatinine, BUN, TG, HDL, LDL, total cholesterol, urinalysis, and hematology), treatment‐related adverse events (TEAEs), and SAEs, were monitored and documented throughout the trial. The results were reported herein following the Consolidated Standards of Reporting Trials reporting guideline.

### 2.10 Statistical analysis

In the oral toxicity rodent studies, data were shown as mean ± SD. For the main study, data including food consumption, body weights, organ weights, blood coagulation, hematology, and blood chemistry were analyzed using the one‐way analysis of variance method followed by Dunnett's test for comparison between groups. For the recovery study, data were analyzed using the Student's *t*‐test for comparison between groups. A probability value of less than 0.05 (*p* < .05) stands for a significant difference between groups. For all other studies, the statistical methods were described in the previous sections.

In the clinical study, specific statistical analyses were indicated for different parameters. The change from baseline refers to the difference between the values measured at a specific time point (week 8) and the initial measurements taken at baseline and was computed using the following formula: Change from Baseline = Value at week 8 − Baseline value. To compare the changes from baseline between different groups (MS‐20 and placebo), we employed the Wilcoxon rank‐sum test to determine if there is a significant difference for each characteristic (Sayde et al., 2020).

## 3 RESULTS

### 3.1 In vitro gene mutation test

To evaluate the mutagenic potential of Symbiota® (MS‐20), an HPRT gene mutation assay was analyzed after a 5‐h treatment with the test solution in nonactivated and S9‐activated systems. In the nonactivated system, CHO‐K1 cells were treated with the test solution at the following 6 doses: 3.125, 6.25, 12.5, 25, 50, and 100 μL/mL. In the S9‐activated system, cells were exposed to the test solution for the following six concentrations: 1.5625, 3.125, 6.25, 12.5, 25, and 50 μL/mL. None of the cells survived at 100 μL/mL in the nonactivated condition (Table 3). When cells were treated with MS‐20 at 50 μL/mL, the relative survival (RS) rate was 16.8% for treatment without S9 activation and 19.2% for treatment with S9 activation. The mutation frequencies were not significantly elevated compared to the negative control with or without metabolic activation for the examined concentrations. All the study criteria were met according to the guideline, and it was concluded that Symbiota® (MS‐20) does not cause a positive response in the in vitro gene mutation assay.

**TABLE 3:** Summary of the results of CHO/HPRT gene mutation assay on Symbiota® (MS‐20).

Treatment (μL/mL)	S9 (−/+)	Cytotoxicity (%)	Mutation frequency (per 10^6^ clonable cells)
0	−	0	0
3.125	−	8.5	11.3
6.25	−	14.9	5.5
12.5	−	16.2	4.1
25	−	17.9	9.5
50	−	83.2	7.3
100	−	100	NA
Positive^a^	−	6.6	97.2
0	+	0	7.6
1.5625	+	12.6	11.3
3.125	+	18.9	3.7
6.25	+	12	11
12.5	+	27.8	16
25	+	45.6	26.2
50	+	80.8	16.6
Positive^b^	+	44	125.3

^a^ Positive control: Methanesulfonic acid, ethyl ester (EMS) at 0.1 μ1/ml.

^b^ Positive control: *N*‐nitroso‐dimethylamine (DMN) at 100 μg/mL.

### 3.2 In vitro chromosomal aberration test

To investigate the effect of Symbiota® (MS‐20) on inducing structural aberrations in the chromosomes of CHO cells, the chromosomal aberrations induced in various stages of the cell cycle and different metabolic conditions were assessed via three test schemes: (1) Scheme I, cells were treated with MS‐20 for 3 h in the medium without S9; (2) Scheme II, cells were treated with MS‐20 for 3 h in the presence of S9; and (3) Scheme III, cells were exposed to MS‐20 for 20 h without S9. Symbiota® was tested at 6.25, 12.5, 25, 50, and 100 μL/mL in all schemes. The concurrent cytotoxicity showed that the survival rates of cells at 100 μL/mL were 31.2%, 59.6%, and 0% in the three schemes, respectively (Table 4), indicating that maximum analyzable concentrations were achieved. The percentages of aberrant cells at the top analyzable concentrations were 0.5%, 2.5%, and 0%. Therefore, it is concluded that treatment with Symbiota® (MS‐20) did not significantly increase the incidence rates of chromosomal aberrations in CHO cells under the tested conditions, regardless of metabolic activation.

**TABLE 4:** Summary of the results of chromosome aberrations of Symbiota® (MS‐20) in CHO cells.

Condition	Treatment (μL/mL)	Duration (h)	S9 (−/+)	Survival (%)^a^	Aberrant cells (%)
Scheme I (‐S9, 3 h)					
Vehicle	0	3	−	100.0	0
6.25	6.25	3	−	91.8	0
12.5	12.5	3	−	65.5	0
25	25	3	−	62.4	0.5
50	50	3	−	48.8	0.5
100	100	3	−	31.2	Too few metaphases
Positive^b^	−	3	−	NA	23
Scheme II (+S9, 3 h)					
Vehicle	0	3	+	100.0	0
6.25	6.25	3	+	99.3	0
12.5	12.5	3	+	90.8	0
25	25	3	+	88.7	0
50	50	3	+	77.3	1
100	100	3	+	59.6	2.5
Positive^c^	−	3	+	NA	18.5
Scheme III (‐S9, 20 h)					
Vehicle	0	20	−	100.0	0
6.25	6.25	20	−	96.2	0
12.5	12.5	20	−	82.6	0
25	25	20	−	58.7	0
50	50	20	−	15.2	Too few metaphases
100	100	20	−	0.0	Too few metaphases
Positive^b^	−	20	−	NA	22.5

^a^ The concurrent cytotoxicity of test product was assessed in duplicate cultures.

^b^ Positive control: Mitomycin C at 1 μM.

^c^ Positive control: Cyclophosphamide at 40 μM.

### 3.3 Mammalian erythrocyte micronucleus test

The clastogenic effect of Symbiota® (MS‐20) was assessed by its ability to induce micronucleated reticulocytes (RETs) in mouse peripheral blood. Vehicle or 5, 10, and 20 mL/kg MS‐20 were orally delivered to the mice via gavage once a day for 2 days. There was no significant difference in the body weight gain for any of the dosing groups compared to the vehicle control group during the study period (not shown). The average proportion of the reticulocyte in the vehicle control animals was 4.2%. All dosing groups at 36 to 48 h after the last administration of MS‐20 did not show a reduction in the RET ratios. The positive control at 48 h after dosing caused a significant 38% decrease in the ratio of RETs. Therefore, MS‐20 did not cause bone marrow toxicity in the testing dose range.

The incidence of micronucleated reticulocytes in the vehicle control at 36–48 h was 0.5 MN (micronuclei)/1000 RETs after the last administration (Table 5). The value for the positive control group was 28.0 MN/1000 RETs at approximately 48 h after treatment. In contrast, the incidence rates for the 5, 10, and 20 mL/kg MS‐20 groups were 1.0, 0.8, and 1.4 MN/1000 RETs, respectively. Therefore, there was no dose–response effect on the micronucleus induction of MS‐20, and there was no significant elevation of the micronucleus frequency in any dose group compared to the negative control.

**TABLE 5:** The frequencies of micronucleated reticulocytes in the peripheral blood of male ICR mice treated with Symbiota® (MS‐20).

Group	Name	Dose (ml/kg)	Individual animal data (MN/1000 RETs)					Mean ± SD (MN/1000 RETs)
1	Negative control^a^	0	0.5	0.5	1	0.5	0	0.5 ± 0.4
2	Low	5	1.5	1	0.5	0.5	1.5	1.0 ± 0.5
3	Middle	10	0	1.5	2	0.5	0	0.8 ± 0.9
4	High	20	0.5	0	1.5	2.5	1.5	1.4 ± 0.9
5	Positive control^b^	–	36	22.5	33	28.5	20	28.0 ± 6.8

^a^ Negative control: Injection grade water.

^b^ Positive control: 1.0 mg/kg mitomycin C.

### 3.4 Acute oral toxicity study in rats

An acute oral toxicity study was conducted to examine any adverse effect of Symbiota® (MS‐20) treatment within 24 h and in the following 14‐day period. Six males and six females were treated per treatment group. There were no mortality and no clinical signs observed in rats treated with MS‐20 via the oral route at dose levels of 5, 10, and 20 mL/kg, followed by 14 days of observation. There were no differences in the body weight or body weight gain observed between MS‐20‐treated and control rats. At necropsy, all rats showed no observable gross lesions. In conclusion, Symbiota® (MS‐20) caused no acute toxic effects in SD rats at the dose level up to 20 mL/kg (Table S1).

### 3.5 Repeated dose 28‐day oral toxicity study in rats

The purpose of this study was to assess the toxicity of Symbiota® (MS‐20) and to determine the No‐Observed‐Adverse‐Effect‐Level (NOAEL) by oral administration of SD rats for 28 days. Eighty rats were randomized into four groups, each consisting of ten males and ten females. The dose levels of MS‐20 were 0 (vehicle control), 1.5 (low), 5 (middle), and 15 (high) milliliters per kilogram of body weight (mL/kg). Examinations were conducted on the rats concerning general demeanor, clinical signs, mortality, food consumption, body weights, total body weight gain, urinalysis, ophthalmologic changes, hematology, serum chemistry, organ weights, organ‐to‐brain weight ratios, and histopathological evaluation. No mortality was found in the control and MS‐20 treated rats during the study period (Table S2). Intermittent salivation, from Study Day 10 and thereafter, was observed in male and female rats in the high‐dose group (15 mL/kg/day) (Table S3). The incidences for males and females were 9/10 and 10/10, respectively. Slight but statistically significant decreases in food consumption were noted at week 2 and week 3 in male rats and at week 2 and week 4 in female rats in the 15 mL/kg/day treatment group (*p* < .05; Table S4). There were no significant changes in the mean body weight (Figure S1), mean body weight gain, ophthalmologic changes (Table S2), urinalysis (Table S5), hematology (Table S6), serum chemistry (Table S7), organ weights and organ‐to‐brain weight ratios (Table S8), as well as any gross changes between the vehicle control and MS‐20‐treated groups (Table S9). In addition, no treatment‐associated histopathological lesion was observed in either control or MS‐20‐treated animals (Table S10). In conclusion, the results of this study suggest that Symbiota® (MS‐20) at doses up to 15 mL/kg/day did not cause adverse effects in SD rats. Therefore, the 28‐day NOAEL for rats ingesting Symbiota® (MS‐20) was found to be 15 mL/kg/day.

### 3.6 Repeated dose 90‐day subchronic oral toxicity study in rats

No adverse effects and no mortality (Tables S11, S12, and S13) were observed in the 90‐day study in the 1.5 (S‐L), 5.0 (S‐M), and 15.0 (S‐H) mL/kg groups for both males and females. All Symbiota® (MS‐20)‐treated groups had similar food consumption (Figure S2; Table S14) compared to the control groups, and there were no significant differences in body weights (Figure S3; Table S15) among the groups receiving MS‐20 when compared to the control groups at any time point during the experiment. No Symbiota®‐related differences were observed in the absolute organ weights and organ‐to‐brain ratios (Table 6) in female rats. The average of brain weights was significantly decreased in the S‐H group of male rats. However, there were no gross or histological abnormalities, and therefore, this change was considered to not be of toxicological significance. Except for that, no significant difference was observed in male rats for organ‐to‐brain weights in comparison with the control group.

**TABLE 6:** 90‐Day subchronic oral toxicity study organ—weights and organ‐to‐brain ratios (mean ± SD).

Group^a^		C	S‐L	S‐M	S‐H	Re‐C	Re‐H
Dose (mL/kg)		0	1.5	5	15	0	15
*Male rats*							
Organ	Unit						
Brain	Weight (g)	2.192 ± 0.071	2.204 ± 0.083	2.155 ± 0.081	2.078 ± 0.029*	2.198 ± 0.044	2.138 ± 0.069
Adrenals	Weight (g)	0.0515 ± 0.0120	0.0505 ± 0.0041	0.0577 ± 0.0118	0.0557 ± 0.0079	0.0458 ± 0.0115	0.0440 ± 0.0073
	Ratio (%)	2.3420 ± 0.5124	2.2967 ± 0.2385	2.6899 ± 0.5957	2.6829 ± 0.3973	2.0770 ± 0.4785	2.0534 ± 0.2948
Heart	Weight (g)	1.480 ± 0.101	1.547 ± 0.125	1.504 ± 0.179	1.410 ± 0.130	1.412 ± 0.061	1.528 ± 0.234
	Ratio	0.674 ± 0.039	0.703 ± 0.055	0.699 ± 0.087	0.679 ± 0.060	0.642 ± 0.020	0.712 ± 0.084
Kidneys	Weight (g)	3.810 ± 0.327	3.947 ± 0.515	3.835 ± 0.440	3.811 ± 0.440	3.650 ± 0.430	3.584 ± 0.734
	Ratio	1.738 ± 0.117	1.793 ± 0.243	1.782 ± 0.203	1.834 ± 0.209	1.658 ± 0.166	1.674 ± 0.290
Liver	Weight (g)	14.818 ± 1.112	15.446 ± 2.197	15.152 ± 1.721	14.447 ± 2.040	14.486 ± 1.677	14.564 ± 2.612
	Ratio	6.761 ± 0.478	7.011 ± 1.001	7.032 ± 0.747	6.951 ± 0.968	6.586 ± 0.698	6.792 ± 0.996
Spleen	Weight (g)	0.658 ± 0.116	0.756 ± 0.122	0.705 ± 0.075	0.706 ± 0.129	0.704 ± 0.106	0.752 ± 0.125
	Ratio	0.299 ± 0.047	0.343 ± 0.057	0.329 ± 0.042	0.340 ± 0.060	0.322 ± 0.049	0.348 ± 0.046
Epididymides	Weight (g)	1.536 ± 0.148	1.560 ± 0.263	1.552 ± 0.162	1.490 ± 0.119	1.572 ± 0.111	1.494 ± 0.157
	Ratio	0.702 ± 0.072	0.707 ± 0.107	0.723 ± 0.091	0.716 ± 0.055	0.712 ± 0.043	0.698 ± 0.068
Pro‐semi vesicle with coa gland	Weight (g)	3.532 ± 0.548	3.384 ± 0.322	3.250 ± 0.404	3.331 ± 0.480	3.858 ± 0.668	3.500 ± 0.245
	Ratio	1.612 ± 0.257	1.538 ± 0.163	1.509 ± 0.199	1.601 ± 0.220	1.756 ± 0.297	1.636 ± 0.100
Testes	Weight (g)	3.452 ± 0.238	3.569 ± 0.164	3.595 ± 0.347	3.524 ± 0.172	3.606 ± 0.305	3.618 ± 0.359
	Ratio	1.575 ± 0.110	1.621 ± 0.091	1.674 ± 0.202	1.697 ± 0.101	1.640 ± 0.114	1.692 ± 0.115
Thymus	Weight (g)	0.256 ± 0.077	0.273 ± 0.069	0.232 ± 0.044	0.230 ± 0.061	0.232 ± 0.038	0.244 ± 0.011
	Ratio	0.116 ± 0.037	0.124 ± 0.032	0.108 ± 0.021	0.111 ± 0.027	0.106 ± 0.018	0.114 ± 0.005
Pituitary gland	Weight (g)	0.0152 ± 0.0014	0.0162 ± 0.0015	0.0161 ± 0.0022	0.0152 ± 0.0023	0.0190 ± 0.0014	0.0204 ± 0.0026
	Ratio (%)	0.6935 ± 0.0604	0.7361 ± 0.0753	0.7477 ± 0.1017	0.7314 ± 0.1119	0.8648 ± 0.0686	0.9530 ± 0.1060
Thyroid gland	Weight (g)	0.0229 ± 0.0032	0.0255 ± 0.0048	0.0258 ± 0.0045	0.0212 ± 0.0036	0.0198 ± 0.0033	0.0222 ± 0.0062
	Ratio (%)	1.0427 ± 0.1230	1.1570 ± 0.2203	1.1985 ± 0.2148	1.0200 ± 0.1730	0.9026 ± 0.1616	1.0358 ± 0.2795
*Female rats*							
Organ	Unit						
Brain	Weight (g)	2.054 ± 0.109	2.065 ± 0.160	2.040 ± 0.088	2.023 ± 0.063	2.026 ± 0.045	1.988 ± 0.095
Adrenals	Weight (g)	0.0722 ± 0.0158	0.0664 ± 0.0070	0.0686 ± 0.0109	0.0721 ± 0.0114	0.0624 ± 0.0060	0.0662 ± 0.0059
	Ratio (%)	3.5257 ± 0.8107	3.2378 ± 0.4402	3.3638 ± 0.5285	3.5707 ± 0.6000	3.0794 ± 0.2831	3.3284 ± 0.2240
Heart	Weight (g)	1.027 ± 0.066	0.954 ± 0.079	0.980 ± 0.069	0.961 ± 0.075	0.952 ± 0.033	0.960 ± 0.022
	Ratio	0.502 ± 0.030	0.464 ± 0.042	0.481 ± 0.039	0.476 ± 0.041	0.470 ± 0.027	0.484 ± 0.025
Kidneys	Weight (g)	2.203 ± 0.172	1.959 ± 0.080	2.171 ± 0.455	2.020 ± 0.184	1.968 ± 0.102	1.892 ± 0.105
	Ratio	1.073 ± 0.054	0.953 ± 0.075	1.063 ± 0.210	1.000 ± 0.094	0.974 ± 0.054	0.952 ± 0.067
Liver	Weight (g)	9.215 ± 0.857	8.447 ± 0.791	8.543 ± 0.807	8.996 ± 0.768	8.386 ± 0.751	8.234 ± 0.398
	Ratio	4.490 ± 0.362	4.111 ± 0.471	4.185 ± 0.299	4.450 ± 0.384	4.138 ± 0.345	4.150 ± 0.306
Spleen	Weight (g)	0.518 ± 0.062	0.466 ± 0.046	0.495 ± 0.054	0.507 ± 0.067	0.464 ± 0.05	0.462 ± 0.058
	Ratio	0.253 ± 0.027	0.227 ± 0.022	0.243 ± 0.030	0.250 ± 0.032	0.230 ± 0.023	0.232 ± 0.036
Ovaries	Weight (g)	0.0858 ± 0.0102	0.0858 ± 0.0155	0.0877 ± 0.0120	0.0855 ± 0.0094	0.0750 ± 0.0120	0.0772 ± 0.0132
	Ratio	4.1826 ± 0.4869	4.1685 ± 0.7773	4.3015 ± 0.6008	4.2347 ± 0.5209	3.6944 ± 0.5360	3.9138 ± 0.8503
Uterus	Weight (g)	0.790 ± 0.253	0.730 ± 0.221	0.686 ± 0.198	0.689 ± 0.295	0.692 ± 0.135	1.024 ± 0.399
	Ratio	0.387 ± 0.137	0.352 ± 0.097	0.336 ± 0.090	0.340 ± 0.137	0.342 ± 0.067	0.518 ± 0.208
Thymus	Weight (g)	0.235 ± 0.090	0.214 ± 0.043	0.219 ± 0.033	0.218 ± 0.064	0.202 ± 0.028	0.216 ± 0.024
	Ratio	0.115 ± 0.044	0.105 ± 0.023	0.109 ± 0.017	0.109 ± 0.033	0.100 ± 0.010	0.106 ± 0.011
Pituitary gland	Weight (g)	0.0198 ± 0.0028	0.0192 ± 0.0045	0.0192 ± 0.0044	0.0196 ± 0.0039	0.0160 ± 0.0020	0.0158 ± 0.0018
	Ratio (%)	0.9638 ± 0.1246	0.9382 ± 0.2361	0.9375 ± 0.1930	0.9684 ± 0.1878	0.7894 ± 0.0950	0.7992 ± 0.1290
Thyroid gland	Weight (g)	0.0196 ± 0.0046	0.0241 ± 0.0049	0.0240 ± 0.0044	0.0206 ± 0.0036	0.0206 ± 0.0054	0.0188 ± 0.0040
	Ratio (%)	0.9576 ± 0.2330	1.1661 ± 0.2136	1.1787 ± 0.2264	1.0186 ± 0.1747	1.0182 ± 0.2679	0.9506 ± 0.2214

*Note*: Data were presented as mean ± SD of 10 animals per group in main study; five animals per group in recovery study.

* Significant difference compared to the control group (*p* < .05).

^a^ C: control; S‐L: Symbiota® low dose group; S‐M: Symbiota® middle dose group; S‐H: Symbiota® high dose group; Re‐C: recovery control group; Re‐H: recovery high dose group. Pro‐semi vesicle with CoA gland: Prostate‐seminal vesicle with coagulating gland.

There were no treatment‐related effects on hematology (Table 7), blood coagulation (Table S16), and urinalysis parameters (Tables S17 and S18). Despite minor differences in the APTT, increases were observed in females treated with middle‐dose MS‐20 compared to controls (Control: 15.38 ± 1.05 s, S‐M: 16.58 ± 1.23 s) but not in males. Nonetheless, the changes in the APTT in the S‐M group were within the acceptable normal physiological range (APTT: 12.23~30.08 s) of reference values (MedGaea Life Sciences Institute); thus, it was not considered to be toxicologically significant. In addition, no significant difference in the PT was observed between all treatment groups and the control groups in the main and recovery studies. No significant differences were found between the control and MS‐20 groups of both sexes in any of the tested clinical chemistry parameters (Table 8). No clinical signs of toxicity, ophthalmologic abnormalities (Table S19), or particular treatment‐related histopathological lesions (Table S20, detailed in Discussion) were noted in the MS‐20‐treated rats of either sex during the study period. In addition, MS‐20 treatment did not interfere with serum thyroid hormones (Table S21). According to the results of a 90‐day subchronic study, after 13 weeks of daily oral dosing of Symbiota® (MS‐20), the NOAEL was 15.0 mL/kg/day in Sprague–Dawley rats.

**TABLE 7:** 90‐Day subchronic oral toxicity study organ—hematology analysis (mean ± SD).

Group^a^	C	S‐L	S‐M	S‐H	Re‐C	Re‐H
Dose (mL/kg)	0	1.5	5	15	0	15
*Male rats*						
Test parameter						
WBC (/μL)	11,432.0 ± 1818.0	9952.0 ± 1520.1	9841.0 ± 1328.0	10,908.0 ± 1775.4	9814.0 ± 919.2	9064.0 ± 1676.5
RBC (10^6^/μL)	9.236 ± 0.694	9.261 ± 0.412	9.34 ± 0.400	9.340 ± 0.538	8.722 ± 0.306	8.906 ± 0.337
HGB (g/dL)	16.41 ± 0.74	15.78 ± 0.66	16.22 ± 0.49	16.33 ± 0.56	15.26 ± 0.35	15.70 ± 0.78
HCT (%)	47.80 ± 1.80	45.50 ± 1.92	46.40 ± 1.49	46.86 ± 2.42	44.16 ± 1.05	45.02 ± 2.77
MCV (fL)	51.97 ± 3.56	49.18 ± 2.07	49.74 ± 1.94	50.29 ± 3.35	50.40 ± 1.87	50.60 ± 3.32
MCH (pg)	17.81 ± 0.76	17.05 ± 0.65	17.39 ± 0.52	17.50 ± 0.82	17.40 ± 0.64	17.64 ± 0.92
MCHC (%)	34.32 ± 0.89	34.69 ± 0.39	34.95 ± 0.45	34.87 ± 0.84	34.56 ± 0.18	34.92 ± 0.54
PLT (10^3^/μL)	1069.4 ± 173.3	1175.5 ± 135.5	1161.9 ± 116.9	1088.4 ± 57.3	1022.4 ± 80.1	1034.8 ± 80.8
MPV (fL)	6.71 ± 0.21	6.77 ± 0.18	6.68 ± 0.19	6.75 ± 0.24	6.76 ± 0.23	7.02 ± 0.25
NEUT (%)	13.62 ± 2.69	14.98 ± 2.09	16.45 ± 2.94	15.54 ± 3.05	13.76 ± 2.96	15.6 ± 2.93
LYMPH (%)	81.32 ± 3.09	79.03 ± 2.41	77.81 ± 3.55	79.29 ± 2.88	81.10 ± 3.00	78.42 ± 2.46
MONO (%)	3.46 ± 0.60	4.20 ± 0.73	3.82 ± 0.65	3.69 ± 0.66	3.92 ± 0.69	4.14 ± 1.17
EOS (%)	1.47 ± 0.60	1.66 ± 0.60	1.81 ± 0.52	1.38 ± 0.45	1.08 ± 0.33	1.76 ± 0.61
BAŞO (%)	0.13 ± 0.05	0.13 ± 0.08	0.11 ± 0.06	0.10 ± 0.07	0.14 ± 0.05	0.08 ± 0.08
RET (%)	2.83 ± 0.29	3.02 ± 0.28	2.96 ± 0.42	2.91 ± 0.32	2.96 ± 0.47	2.88 ± 0.25
*Female rats*						
Test parameter						
WBC (/μL)	8155.0 ± 845.6	8158.0 ± 829.9	8427.0 ± 956.5	8251.0 ± 859.6	8450.0 ± 1328.3	8874.0 ± 1253.3
RBC (10^6^/μL)	8.188 ± 0.280	8.272 ± 0.294	8.583 ± 0.313	8.465 ± 0.530	8.086 ± 0.292	8.014 ± 0.397
HGB (g/dL)	15.08 ± 0.40	15.32 ± 0.72	15.84 ± 0.44	15.50 ± 0.81	15.08 ± 0.42	14.74 ± 0.38
HCT (%)	43.34 ± 1.02	44.41 ± 2.18	45.49 ± 0.92	44.62 ± 2.10	43.80 ± 1.19	42.46 ± 1.28
MCV (fL)	52.96 ± 1.64	53.67 ± 1.24	53.06 ± 1.86	52.75 ± 1.24	54.22 ± 2.17	53.02 ± 1.01
MCH (pg)	18.41 ± 0.57	18.53 ± 0.42	18.48 ± 0.57	18.34 ± 0.44	18.64 ± 0.7	18.40 ± 048
MCHC (%)	34.80 ± 0.36	34.50 ± 0.36	34.82 ± 0.56	34.74 ± 0.54	34.42 ± 0.45	34.72 ± 0.30
PLT (10^3^/μL)	1120.7 ± 159.5	1075.7 ± 112.6	1131.4 ± 148.0	1057.6 ± 89.2	1073.4 ± 98.4	1035.4 ± 102.2
MPV (fL)	6.88 ± 0.29	6.99 ± 0.25	7.02 ± 0.26	6.93 ± 0.14	7.08 ± 0.28	7.06 ± 0.19
NEUT (%)	10.79 ± 3.52	10.81 ± 2.47	10.11 ± 2.81	12.22 ± 1.73	11.14 ± 2.68	12.44 ± 2.24
LYMPH (%)	84.01 ± 4.05	84.30 ± 2.67	85.28 ± 3.07	83.29 ± 1.85	84.60 ± 2.96	82.40 ± 2.42
MONO (%)	3.54 ± 0.91	3.33 ± 0.72	3.02 ± 0.51	3.08 ± 0.85	3.26 ± 0.63	3.72 ± 0.47
EOS (%)	1.54 ± 0.26	1.41 ± 0.40	1.50 ± 0.48	1.33 ± 0.51	0.94 ± 0.24	1.30 ± 0.46
BAŞO (%)	0.12 ± 0.09	0.15 ± 0.12	0.09 ± 0.07	0.08 ± 0.10	0.06 ± 0.09	0.14 ± 0.13
RET (%)	3.64 ± 0.39	3.48 ± 0.39	3.57 ± 0.35	3.74 ± 0.36	3.42 ± 0.33	3.20 ± 0.37

*Note*: Data were presented as mean ± SD of 10 animals per group in main study; five animals per group in recovery study.

Abbreviations: BASO, Basophil; EOS, Eosinophil; HCT, Hematocrit; HGB, Hemoglobin; LYMPH, Lymphocyte; MCH, Mean corpuscular hemoglobin; MCHC, Mean corpuscular hemoglobin concentration; MCV, Mean corpuscular volume; MONO, Monocyte; MPV, Mean platelet volume; NEUT, Neutrophil; PLT, Platelet; RBC, Red blood cell; RET, Reticulocyte; WBC, White blood cell.

^a^ C: control; S‐L: Symbiota® low dose group; S‐M: Symbiota® middle dose group; S‐H: Symbiota® high dose group; Re‐C: recovery control group; Re‐H: recovery high dose group.

**TABLE 8:** 90‐Day subchronic oral toxicity study organ—clinical chemistry analysis (mean ± SD).

Main study									
Group^a^		C		S‐L		S‐M		S‐H	
Dose (mL/kg)		0		1.5		5		15	
Sex		Male	Female	Male	Female	Male	Female	Male	Female
*Test parameters*									
ALT	U/L	29.4 ± 5.7	24.0 ± 5.0	28.7 ± 6.6	25.7 ± 10.0	27.3 ± 2.7	20.7 ± 2.4	27.6 ± 4.6	29.2 ± 16.0
AST	U/L	108.2 ± 23.5	105.1 ± 15.0	93.6 ± 17.2	99.8 ± 29.7	105.8 ± 27.3	85.3 ± 12.9	97.1 ± 15.7	109.2 ± 23.7
ALP	U/L	75.3 ± 27.4	40.6 ± 12.0	77.0 ± 8.5	38.4 ± 12.0	71.2 ± 10.3	3.82 ± 28.9	83.0 ± 27.0	45.9 ± 9.7
TBIL	mg/dL	0.131 ± 0.019	0.141 ± 0.019	0.128 ± 0.021	0.131 ± 0.023	0.123 ± 0.018	0.141 ± 0.026	0.145 ± 0.039	0.136 ± 0.019
GGT	U/L	0.59 ± 0.22	0.50 ± 0.23	0.55 ± 0.16	0.41 ± 0.26	0.52 ± 0.28	0.60 ± 0.20	0.73 ± 0.43	0.50 ± 0.25
TP	g/dL	6.85 ± 0.37	7.27 ± 0.42	6.74 ± 0.24	7.53 ± 0.41	6.66 ± 0.24	7.18 ± 0.48	6.69 ± 0.43	7.10 ± 0.43
ALB	g/dL	3.56 ± 0.16	4.00 ± 0.19	3.54 ± 0.13	4.06 ± 0.46	3.56 ± 0.20	4.09 ± 0.29	3.47 ± 0.28	3.95 ± 0.21
GLO	g/dL	3.29 ± 0.22	3.28 ± 0.23	3.20 ± 0.19	3.47 ± 0.32	3.10 ± 0.12	3.12 ± 0.18	3.22 ± 0.35	3.17 ± 0.30
AMY	U/L	476.3 ± 51.4	354.3 ± 67.8	501.3 ± 86.9	358 ± 73.0	511.5 ± 70.0	362.6 ± 122.1	421.4 ± 60.8	390.8 ± 76.8
BUN	mg/dL	18.25 ± 1.79	19.57 ± 2.50	17.40 ± 2.16	18.56 ± 1.62	19.79 ± 2.03	16.51 ± 2.61	17.54 ± 1.71	18.32 ± 2.71
CRE	mg/dL	0.460 ± 0.052	0.514 ± 0.061	0.495 ± 0.069	0.470 ± 0.049	0.452 ± 0.066	0.452 ± 0.056	0.467 ± 0.052	0.475 ± 0.050
LDH	U/L	456.7 ± 50.0	459.2 ± 41.2	461.3 ± 77.9	538.7 ± 195.1	453.4 ± 50.3	458.4 ± 41.2	453.1 ± 50.5	434.0 ± 30.6
CPK	U/L	349.3 ± 75.6	446.1 ± 47.4	344.1 ± 33.9	436.6 ± 50.8	349.8 ± 56.8	391.9 ± 72.8	347.9 ± 50.1	455.6 ± 27.2
GLU	mg/dL	143.0 ± 24.3	122.4 ± 12.2	135.9 ± 25.6	136.3 ± 11.2	129.1 ± 22.0	133.1 ± 19.0	125.7 ± 19.4	131.4 ± 22.0
TC	mg/dL	72.4 ± 13.8	91.8 ± 15.7	72.3 ± 14.1	86.6 ± 16.1	74.4 ± 10.6	85.5 ± 11.5	71.7 ± 16.5	91.8 ± 15.4
TG	mg/dL	59.0 ± 22.6	63.4 ± 13.2	69.0 ± 39.9	49.7 ± 10.6	56.6 ± 24.3	49.9 ± 13.2	61.0 ± 22.9	50.4 ± 17.6
Na	mmol/L	149.6 ± 2.1	145.9 ± 1.3	149.0 ± 2.4	146.5 ± 1.1	149.3 ± 1.2	145.3 ± 1.2	148.6 ± 1.8	144.9 ± 1.9
K	mmol/L	7.24 ± 1.33	7.96 ± 0.60	7.82 ± 1.61	7.42 ± 0.81	6.86 ± 0.60	7.43 ± 0.66	7.04 ± 1.07	8.25 ± 1.29
Cl	mmol/L	97.2 ± 2.1	98.7 ± 1.3	98.6 ± 1.3	100.3 ± 2.0	98.3 ± 1.3	99.5 ± 1.3	96.0 ± 1.9	100.0 ± 1.5
Ca	mg/dL	11.20 ± 0.51	11.57 ± 0.36	11.03 ± 0.68	11.94 ± 0.71	11.02 ± 0.25	11.63 ± 0.37	10.99 ± 0.38	11.48 ± 0.55
P	mg/dL	13.25 ± 0.62	12.00 ± 2.13	12.84 ± 1.26	10.38 ± 1.07	12.75 ± 0.86	10.63 ± 0.96	13.37 ± 1.41	11.01 ± 1.27
HDL‐C	mg/dL	41.2 ± 7.2	57.7 ± 18.5	36.7 ± 10.6	54.7 ± 15.0	43.6 ± 7.4	54.2 ± 7.3	39.6 ± 10.9	59.6 ± 12.3
LDL‐C	mg/dL	19.40 ± 8.41	21.42 ± 8.25	21.80 ± 10.32	21.96 ± 7.28	19.48 ± 5.58	21.32 ± 6.14	19.90 ± 7.10	22.12 ± 11.88
TBA	μM	9.57 ± 4.49	12.01 ± 6.18	12.21 ± 6.79	10.61 ± 5.09	12.03 ± 5.22	13.15 ± 6.66	13.04 ± 6.10	15.69 ± 5.10

Recovery study					
Group^a^		Re‐C		Re‐H	
Dose (mL/kg)		0		15	
Sex		Male	Female	Male	Female
*Test parameters*					
ALT	U/L	30.0 ± 4.8	24.8 ± 3.8	29.4 ± 4.9	26.8 ± 7.2
AST	U/L	78.6 ± 6.9	77.2 ± 6.6	71.2 ± 11.2	82.8 ± 18.4
ALP	U/L	65.4 ± 11.4	27.4 ± 8.2	81.2 ± 17.4	34.6 ± 8.9
TBIL	mg/dL	0.130 ± 0.007	0.150 ± 0.019	0.112 ± 0.027	0.132 ± 0.013
GGT	U/L	0.12 ± 0.08	0.36 ± 0.11	0.24 ± 0.13	0.32 ± 0.19
TP	g/dL	6.44 ± 0.57	7.16 ± 0.32	6.38 ± 0.25	7.00 ± 0.32
ALB	g/dL	3.34 ± 0.17	4.00 ± 0.16	3.38 ± 0.16	3.90 ± 0.23
GLO	g/dL	3.10 ± 0.41	3.18 ± 0.24	3.00 ± 0.19	3.12 ± 0.22
AMY	U/L	521.8 ± 118.8	447.6 ± 184.2	512.2 ± 87.8	301.6 ± 42.5
BUN	mg/dL	17.34 ± 2.22	16.10 ± 1.86	16.06 ± 0.62	18.20 ± 2.31
CRE	mg/dL	0.378 ± 0.019	0.420 ± 0.041	0.368 ± 0.015	0.420 ± 0.092
LDH	U/L	462.0 ± 48.8	468.6 ± 26.4	432.4 ± 60.9	460.6 ± 30.3
CPK	U/L	341.2 ± 55.8	440.0 ± 22.7	344.0 ± 50.7	462.6 ± 35.0
GLU	mg/dL	165.6 ± 41.4	174.2 ± 14.9	176.6 ± 13.6	171.8 ± 40.8
TC	mg/dL	85.4 ± 18.6	94.6 ± 7.7	76.4 ± 14.0	89.0 ± 13.5
TG	mg/dL	48.0 ± 23.1	73.0 ± 13.4	46.8 ± 18.5	58.6 ± 21.5
Na	mmol/L	146.4 ± 0.9	146.0 ± 1.0	148.2 ± 1.9	145.6 ± 0.5
K	mmol/L	7.82 ± 0.65	6.62 ± 0.41	6.90 ± 1.02	6.56 ± 0.76
Cl	mmol/L	100.2 ± 1.6	101.4 ± 2.6	102.0 ± 0.7	103.8 ± 1.5
Ca	mg/dL	11.32 ± 0.42	11.94 ± 0.24	11.68 ± 0.33	11.66 ± 0.60
P	mg/dL	12.62 ± 1.08	7.66 ± 1.17	12.52 ± 1.30	7.08 ± 0.85
HDL‐C	mg/dL	51.0 ± 20.6	59.4 ± 6.5	43.6 ± 13.7	57.4 ± 11.5
LDL‐C	mg/dL	24.80 ± 10.88	20.60 ± 6.10	23.44 ± 3.81	19.88 ± 5.28
TBA	μM	12.02 ± 6.86	12.28 ± 3.23	17.78 ± 4.12	15.28 ± 3.32

*Note*: Data were presented as mean ± SD of 10 animals per group in main study; five animals per group in recovery study.

Abbreviations: ALB, Albumin; ALP, Alkaline phosphatase; ALT, Alanine aminotransferase; AMY, Amylase; AST, Aspartate aminotransferase; BUN, Blood urea nitrogen; Ca, Calcium; Cl, Chloride; CPK, Creatine phosphokinase; CRE, Creatinine; GGT, I‐glutamyl transpeptidase; GLO, Globulin; GLU, Glucose; HDL‐C, high‐density lipoprotein cholesterol; K, Potassium; LDH, Lactate dehydrogenase; LDL‐C, low‐density lipoprotein cholesterol; Na, Sodium; P, Inorganic phosphorus; TBA, total bile acid; TBIL, Total bilirubin; TC, Total cholesterol; TG, Triglyceride; TP, Total protein.

^a^ C: control; S‐L: Symbiota® low dose group; S‐M: Symbiota® middle dose group; S‐H: Symbiota® high dose group; Re‐C: recovery control group; Re‐H: recovery high dose group.

### 3.7 Clinical study with healthy volunteers

A total of 101 subjects signed an informed consent form, and one withdrew consent (Figure 1). The remaining 100 subjects were randomized, 50 to the Symbiota® (MS‐20) group and 50 to the placebo group. Subject demographic data are presented in Table 9. The average age of the participants was 41.8 years in the MS‐20 group and 38.6 years in the placebo group. The mean height, weight, BMI, and sex ratios were similar between the two groups.

**TABLE 9:** Patient demographics.

Characteristic^a^	Patient group			*p* value
	All (*n* = 100)	MS‐20 (*n* = 50)	Placebo (*n* = 50)	
Baseline patient characteristics				
Age, year	40.2 (10)	41.8 (10.9)	38.6 (8.7)	.06^‡^
Sex				.52^§^
Male (%)	32 (32%)	18 (36%)	14 (28%)	–
Female (%)	68 (68%)	32 (64%)	36 (72%)	–
Height (SD), cm	163.4 (8.8)	163.4 (8.6)	163.5 (9)	.90^‡^
Weight (SD), kg	62.5 (14)	60.9 (11.6)	64.1 (16)	.29^‡^
BMI	23.2 (3.6)	22.6 (2.7)	23.7 (4.3)	.18^‡^
Smoking, number (Y/N/Quit)	8/91/1	4/45/1	4/46/0	
Systolic blood pressure, mmHg	116.5 (17.9)	117.9 (16.6)	115.1 (19.2)	.18^‡^
Diastolic blood pressure, mmHg	70.4 (11.9)	69.9 (9.6)	70.8 (13.9)	.79^‡^

^‡^ Two sample *t*‐test, statistical significance was determined if *p* < .05.

^§^ Chi‐squared test, statistical significance was determined if *p* < .05.

^a^ Unless otherwise indicated, data are expressed as mean (SD).

To assess the safety and tolerability of MS‐20, the occurrence of treatment‐emergent adverse events (TEAEs) and the changes from baseline in vital signs, physical examination, and laboratory test results at week 8 were analyzed. Overall, three subjects (3%, 3/100) reported 3 TEAEs. These included two subjects (4%, 2/50, one patient with grade 1; one patient with grade 2, according to CTCAE v5.0) in the MS‐20 group, and one subject (2%, 1/50, grade 2) in the placebo group. All three TEAEs involved abnormal serum AST. No severe TEAEs were reported in either the MS‐20 or the placebo group. The majority of shifts from baseline in chemistry, hematology, and coagulation parameters were similar with no statistical significance between the treatment groups (Table 10). Only the changes from baseline in hs‐CRP, a marker for cardiovascular disease (CVD) risk or acute inflammation, were mildly, but significantly, decreased in the MS‐20 group when compared to the placebo group. Overall, the safety profile of Symbiota® (MS‐20) was acceptable and comparable to that of the placebo.

**TABLE 10:** Patient characteristics at baseline and after the 8‐week intake of Symbiota® (MS‐20) or Placebo.

Characteristic^a^	MS‐20 (*n* = 50)		Placebo (*n* = 50)		*p* value^§^
	Baseline	Week 8	Baseline	Week 8	
Homocysteine, μmol/L	8.8 [7.3, 10.7]	8.3 [6.9, 10.2]	8.9 [7.4, 10.8]	8.2 [7.3, 10.1]	.96
Fasting glucose, mg/dL	90 [84.3, 95.8]	89 [85, 96.8]	89 [86, 93]	88.5 [83.3, 92]	.17
AST, U/L	17 [15, 20]	16.5 [15, 21.8]	16.5 [15, 21]	16 [14, 19]	.05
Creatinine, mg/dL	0.7 [0.6, 0.9]	0.7 [0.6, 0.9]	0.7 [0.6, 0.8]	0.7 [0.6, 0.8]	.40
Cholesterol, mg/dL	198 [176.3, 220.5]	203.5 [174, 225.8]	186.5 [169, 215.8]	183 [168, 214.3]	.99
TG, mg/dL	79 [65.3, 102.3]	81.5 [60.3, 110.5]	85.5 [64.5, 120.3]	75 [63, 122.5]	.46
LDL, mg/dL	119.5 [96, 134.8]	115 [91.3, 135.8]	112.5 [94.3, 142.3]	117.5 [95.3, 135]	.61
HDL, mg/dL	61 [50.3, 71.8]	58 [48.5, 70.8]	54.5 [48, 62]	53 [49, 61]	.87
hs‐CRP^b^, mg/dL	0.07 [0.04, 0.14]	0.06 [0.03, 0.09]	0.07 [0.04, 0.17]	0.07 [0.04, 0.14]	.04*
RBC, M/μL	4.7 [4.4, 5.3]	4.8 [4.4, 5.2]	4.8 [4.4, 5.3]	4.8 [4.4, 5.2]	.18
Hb, g/dL	13.8 [13, 15]	14 [12.9, 14.9]	13.6 [12.9, 14.4]	13.3 [12.7, 14.4]	.30
HCT, %	41.2 [39.8, 44.5]	42.3 [39.6, 45]	41.5 [39.5, 43.8]	40.4 [38.7, 43.5]	.09
MCV, fL	89.4 [86.4, 91.9]	88.7 [85.9, 91.9]	87.9 [83.2, 90.7]	86.8 [83.4, 90.7]	.66
MCH, pg	29.8 [28.7, 30.5]	29.8 [28.7, 30.5]	29.2 [26.3, 30.2]	29.1 [26.2, 30.1]	.65
MCHC, g/dL	33.1 [32.4, 33.6]	33.1 [32.1, 33.8]	32.7 [31.8, 33.5]	33.1 [31.8, 33.6]	.25
Platelet, K/μL	264 [235.5, 302]	262 [227, 291.8]	243.5 [218.5, 299.8]	240 [215, 293]	.80
RDW‐CV, %	12.7 [12.1, 13.1]	12.6 [12.1, 13.1]	12.8 [12.2, 14.3]	12.7 [12.1, 13.7]	.10
WBC, K/μL	5.4 [4.6, 6.4]	5.4 [4.6, 6.3]	5.5 [4.8, 6.6]	5.9 [4.3, 6.8]	.95

*Note*: M: 10^6^ cells; K: 10^3^ cells.

^§^ Statistical significance was determined by Wilcoxon rank‐sum test to compare the changes from baseline between groups (*p < .05).

^a^ All values are expressed as median [Q1, Q3].

^b^ Only data above the detection limit (0.02 mg/dL) are included into statistics. MS‐20 (*n* = 46); Placebo (*n* = 47).

## 4 DISCUSSION

The present study offers a complete assessment of the safety of the proprietary product, Symbiota® (MS‐20), including in vitro and in vivo toxicological studies, as well as a human clinical study. To evaluate any genetic toxicity effect of the product, in vitro *hprt* gene mutation assays and chromosome aberration assays were conducted. All positive and negative controls were in place and aligned with the assay criteria. No positive responses were observed in these assays with or without the metabolic activation by the S9 system. Additionally, there was no increased incidence of micronucleated reticulocytes with the highest dose of 20 mL/kg in the in vivo study. Therefore, Symbiota® (MS‐20) does not elicit mutagenic and clastogenic effects according to the results of these studies.

For animal studies, increased salivation was observed in animals treated with the high dose (15 mL/kg/day) in the 28‐day repeated dose study. This clinical sign was also observed in other oral gavage studies and could be attributed to the taste of the test article or other technical reasons instead of treatment‐related toxicity (Eichenbaum et al., 2011; Kim et al., 2003; Yu et al., 2013). In the present study, no increased salivation was seen in any dosing group from the longer 90‐day repeated dose study. Therefore, it is concluded that salivation is not a clinically significant change caused by the treatment. Notably, histopathological lesions (Berridge et al., 2016; Frazier et al., 2012; Renne et al., 2009), including multifocal and focal interstitial mononuclear cell infiltration in the harderian gland, coagulating gland, and kidney; focal mononuclear cell infiltration in the heart; focal calculus, multifocal tubular cast, focal tubular infarct, and multifocal tubular mineralization in the kidneys; and focal macrophage aggregation in the lungs, were randomly found in the control and Symbiota® groups in the 90‐day repeated dose study. In addition, focal hemorrhage and inflammation in the submucosa of the stomach were incidental lesions in one male rat. These observations may be an artifact associated with the gavage. A high incidence of mineralized renal tubules at the corticomedullary junction was reported in female Sprague–Dawley, Wistar, and Fischer 344 rats, suggesting a strong influence of female sex hormones on the development of the lesion (Rao, 2002). All lesions listed above were not considered to be related to the treatment. Overall, there were no positive correlations between the degree and incidence rate of the histological changes between the Symbiota® and control groups. Based on the measured endpoints in the 90‐day subchronic study (OECD TG 408), we concluded that Symbiota® (MS‐20) does not cause any adverse effects on endocrine, immunological, or reproductive organs.

Symbiota® (MS‐20) was well tolerated at 20 mL/kg in the acute toxicity study in rats, which is equivalent to 150‐fold over the daily recommended use level (8 mL for a 60‐kg adult, which is 0.133 mL/kg). Notably, the NOAEL dose of 15 mL/kg established from the 28‐ and 90‐day repeated dose oral toxicity studies provides a safety margin of 113‐fold over the recommended daily dose. Even when a dosage conversion factor of 6.17 for the body surface area difference between rats and humans is considered, there is still a corresponding safety margin of 18‐fold (i.e., equivalent to 2.43 mL/kg in humans). Moreover, the outcomes of the clinical trial reported here further substantiated the safety of a daily intake of 8 mL of Symbiota® in healthy adults. Thus, the product is safe and well tolerated at the intended levels, supported by substantial scientific evidence.

In the 90‐day oral toxicity study in rats, Symbiota® (MS‐20) treatment did not influence the hematology profile (WBCs, RBCs, eosinophils, monocytes, basophils, neutrophils, and lymphocytes) or the immune organ weights (thymus and spleen). LDH, a general indicator of acute or chronic inflammation, was also not elevated. Furthermore, in the clinical study with healthy volunteers present in this article, there was no influence on the hematology profile when Symbiota® (MS‐20) was administered for 8 weeks. All these findings showed that Symbiota® has no significant toxicity to immune cells, and it does not affect the overall inflammatory index of the organism or the balance among immune cell populations. Nonetheless, FSP have been shown to have immune‐modulating properties in short‐term use in both cell culture and animal studies, where elevated activities of NK cells were observed (Chin et al., 2014). A similar phenomenon is also found in other GRAS ingredients that claim to have immunomodulatory functions. The activity of EpiCor (Beta‐1,3/1,6‐glucans from *Saccharomyces cerevisiae*) was shown to induce the activation of NK cells only 2 h after consumption in a clinical trial (Jensen et al., 2011). In another GRAS case, that is, *Spirulina platensis*, NK functions probed by IFN‐gamma secretion and cytolysis were boosted after the supplementation of *Spirulina* in >50% of subjects (Hirahashi et al., 2002). Therefore, consumption of Symbiota® (MS‐20) and such GRAS ingredients may provide a rapid and transient effect on the activation status of specific lymphocyte subsets with no harmful risk to immune organs and overall health.

Soy‐derived foods, like soymilk, soy sauce, natto, tempeh, miso, and tofu, have been widely consumed worldwide in recent decades (Messina et al., 2022), and for hundreds or thousands of years in Asian countries. Soy and its major components, isoflavones, have shown medicinal benefits, including protecting against inflammation, obesity, diabetes, CVDs, neurodegenerative disorders, and osteoporosis (Goh et al., 2022; Mir et al., 2022). However, the weak hormone effects and reproductive concerns of soy and isoflavones are still under debate. The Nordic Council of Ministers report showed that there was no association between precocious puberty and dietary soy or isoflavone consumption in girls and boys. Additionally, daily soy or isoflavone exposure was not found to be associated with breast cancer risk in girls and boys (Bredsdorff et al., 2020). A meta‐analysis indicated no significant difference between isoflavone exposure and reproductive hormone levels in men (Reed et al., 2021). Furthermore, soy food intake of men was not associated with fertilization in vitro (Mínguez‐Alarcón et al., 2015). The European Food Safety Authority (EFSA) reported that no association was found between isoflavone intake and uterine cancer or thyroid function. In the same report, the EFSA also concluded that no significant association was observed between isoflavone consumption and mammographic density, which is associated with breast cancer (Boyd, 2013; EFSA Panel on Food Additives Nutrient Sources added to Food, 2015; Vachon et al., 2015). Of particular note, Symbiota® contains approximately 18 mg/100 g of total isoflavones (genistein, daidzein, and glycitein), which is comparable to other soy‐based foods (Table 2). In approving the health claim of soy proteins for coronary heart disease in 1999, the US FDA established 25 g/day soy proteins as the threshold intake for cholesterol reduction (Stein, 2000). Isoflavone concentrations were reported to range from 0.6 to 2 mg per gram in various sources of soy proteins (Sacks et al., 2006). Thus, the amount of isoflavones (~1.44 mg) for the suggested level of Symbiota® (i.e., 8 mL/day) is much lower than the estimated isoflavone content (16 to 50 mg) in 25 g soy proteins that have been determined to be safe by FDA. Overall, consumption of Symbiota® (MS‐20) at the recommended levels should not incur any health concerns allegedly associated with isoflavones due to a limited amount of exposure.

Symbiota® (MS‐20) products were first introduced as foods and dietary supplements in Taiwan under the tradename, Soy young® or young®, in 2000. These products have been granted three “Health Food” certificates (Health Food no. A00062) from the Taiwan FDA (TFDA) for the following functions: (1) modulating immunity; (2) regulating the blood lipid profile; and (3) improving gastrointestinal functions. In 2011, MS‐20 was approved by the TFDA as a new Traditional Chinese Medicine (TCM) drug (tradename: Chemo young®) that could function as a chemotherapy adjuvant (Chi et al., 2014). Its indication is to ameliorate fatigue and appetite loss in cancer patients who are receiving chemotherapy. In a clinical study, the safety profiles were similar between chemotherapy plus MS‐20 and chemotherapy alone, with improved QoL in the chemotherapy plus MS‐20 group (Chi et al., 2014). Furthermore, postmarketing surveillance of “Chemo young® oral solution,” at the dose of 4 mL twice daily, was conducted in Taiwan for 5 years (from 2012 to 2016) after the approval as a chemotherapy adjuvant, and no adverse events were reported (an official submission to the regulatory authority). Moreover, the product has been exported to Macau SAR since 2018. Based on 20‐year sales and use experiences, Symbiota® products have established a long history of good safety, which is also evidenced by the current study.

This study has some limitations. First, the clinical trial is a single center study, and the source of participants might be limited and lack diversity. Second, female participants comprised 68% of overall recruited subjects. Therefore, sex bias should be considered when evaluating the results. Besides the aforementioned caveats, the general acceptance and compliance of the interventions were good as only one eligible subject refused to join the trial and all allocated participants completed the course of treatment. In addition, the safety profiles reported in this study and from the previous clinical trial support the applications of Symbiota® products for both general healthy subjects and cancer patients who are receiving chemotherapy. Fermented soybean foods have been widely consumed in different parts of world, especially in Asia, and they are also gaining popularity in western counties for the demand of plant‐based food products. However, there is a lack of systemic studies on their health benefits and potential harms. This study provides such a reference for evaluating potential adverse effects of newly developed fermented products on the general population.

## 5 CONCLUSION

The present article provided a comprehensive safety assessment through the battery of guideline‐compliant nonclinical studies and a placebo‐controlled clinical trial to support the safety of Symbiota® (MS‐20) consumption as a nutritional and functional ingredient in selected foods for the general population and in specialized diets for the individuals with certain needs or diseases (e.g. cancer patients) within the proposed use levels.

## AUTHOR CONTRIBUTIONS

**Chien‐Min Hung:** Conceptualization (equal); writing – original draft (lead); writing – review and editing (lead). **Wen‐Cheng Chu:** Conceptualization (supporting); writing – original draft (supporting). **Wen‐Yen Huang:** Conceptualization (supporting); writing – original draft (supporting). **Pei‐Jung Lee:** Conceptualization (supporting); writing – original draft (supporting). **Wen‐Chih Kuo:** Conceptualization (supporting); writing – original draft (supporting). **Cheng‐Yu Hou:** Data curation (equal). **Chia‐Chun Yang:** Data curation (equal). **Ai‐Jen Yang:** Methodology (equal). **Wei‐Kai Wu:** Investigation (equal). **Ming‐Liang Kuo:** Supervision (equal). **Ming‐Shiang Wu:** Investigation (lead). **Wan‐Jiun Chen:** Conceptualization (equal); supervision (equal); writing – original draft (equal); writing – review and editing (equal).

## FUNDING INFORMATION

The sponsor was responsible for designing the study and preparing the manuscript. The sponsor had no role in the conduct of the study, collection, management, analysis, and interpretation of the data.

## CONFLICT OF INTEREST STATEMENT

The authors declared the following potential conflicts of interest: WJC, CMH, WCC, WYH, PJL, WCK, CYH, and MLK are employed by Microbio Co., Ltd., and CCY is employed by Microbio (Shanghai) Biotech Company.

## ETHICS STATEMENT

All subjects gave their written informed consent for inclusion before they participated in the study. The study was conducted in accordance with the Declaration of Helsinki, and the protocol was approved by the Ethics Committee of National Taiwan University Hospital (Study Number: 201903096MIPB). For all animal studies, the protocols were approved by the IACUC.

## Supporting information


Data S1

## Data Availability

The data that support the findings of this study are available from the corresponding author upon reasonable request.
