# Supplementary material for: Safety assessment of a proprietary fermented soybean solution, Symbiota®, as an ingredient for use in foods and dietary supplements: Non‐clinical studies and a randomized trial
Source: Food Sci Nutr. 2024 Jan 8;12(4):2346–63. doi: 10.1002/fsn3.3921 (PMC11016383; doi:10.1002/fsn3.3921)
Supplement: Supplementary file 1 — Data S1 [file FSN3-12-2346-s001.docx]

**Supplementary Materials**

**Safety Assessment of a Proprietary Fermented Soybean Solution, Symbiota^®^, as an Ingredient for Use in Foods and Dietary Supplements: Non-clinical Studies and a Randomized Trial**

Chien-Min Hung^1^, Wen-Cheng Chu^1^, Wen-Yen Huang^1^, Pei-Jung Lee^1^, Wen-Chih Kuo^1^, Cheng-Yu Hou^1^, Chia-Chun Yang^1,2^, Ai-Jen Yang^1^, Wei-Kai Wu^3,4^, Ming-Liang Kuo^1^, Ming-Shiang Wu^4,5^, Wan-Jiun Chen^1*^

^1^ Microbio Co., Ltd, Taipei, Taiwan

^2^ Microbio (Shanghai) Biotech Company, Shanghai, China

^3^ Department of Medical Research, National Taiwan University Hospital, Taipei, Taiwan

^4^ Department of Internal Medicine, National Taiwan University Hospital, Taipei, Taiwan

^5^ Department of Internal Medicine, National Taiwan University College of Medicine, Taipei, Taiwan

* Correspondence: WanJiun.Chen@microbio.com.tw

- Figure S1~S3 (color)
- Table S1~S21


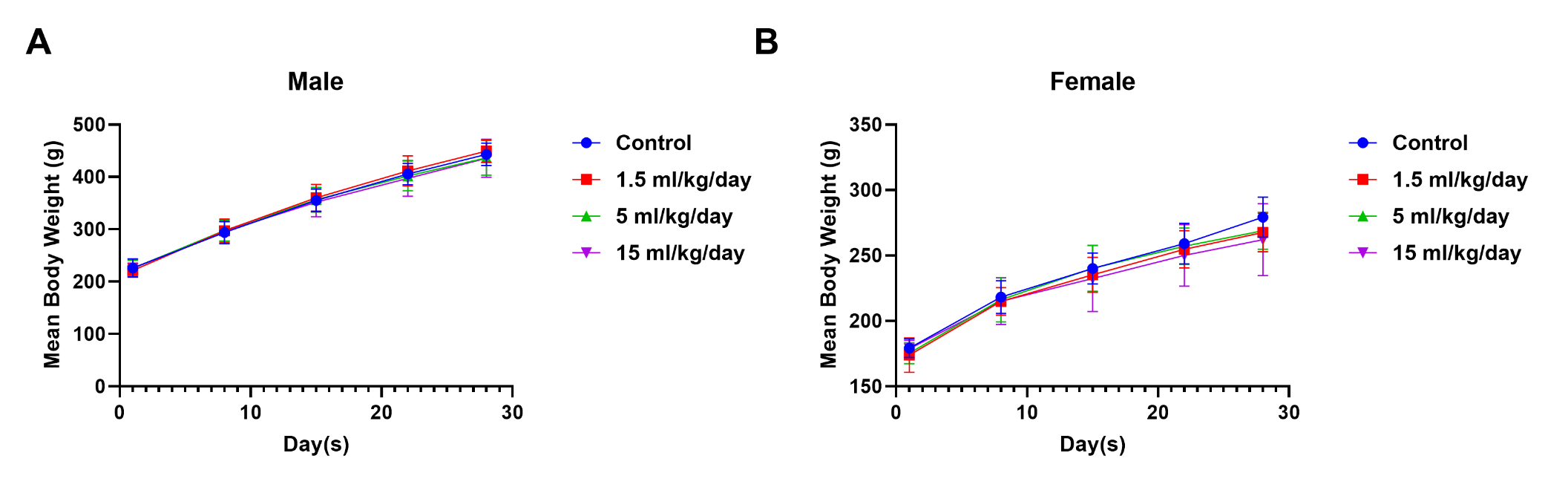


**Figure S1. The growth curve (mean body weight) of 28-Day Subacute Oral Toxicity Study**

(A) Male rats (n=10 for all groups); (B) Female rats (n=10 for all groups)

**
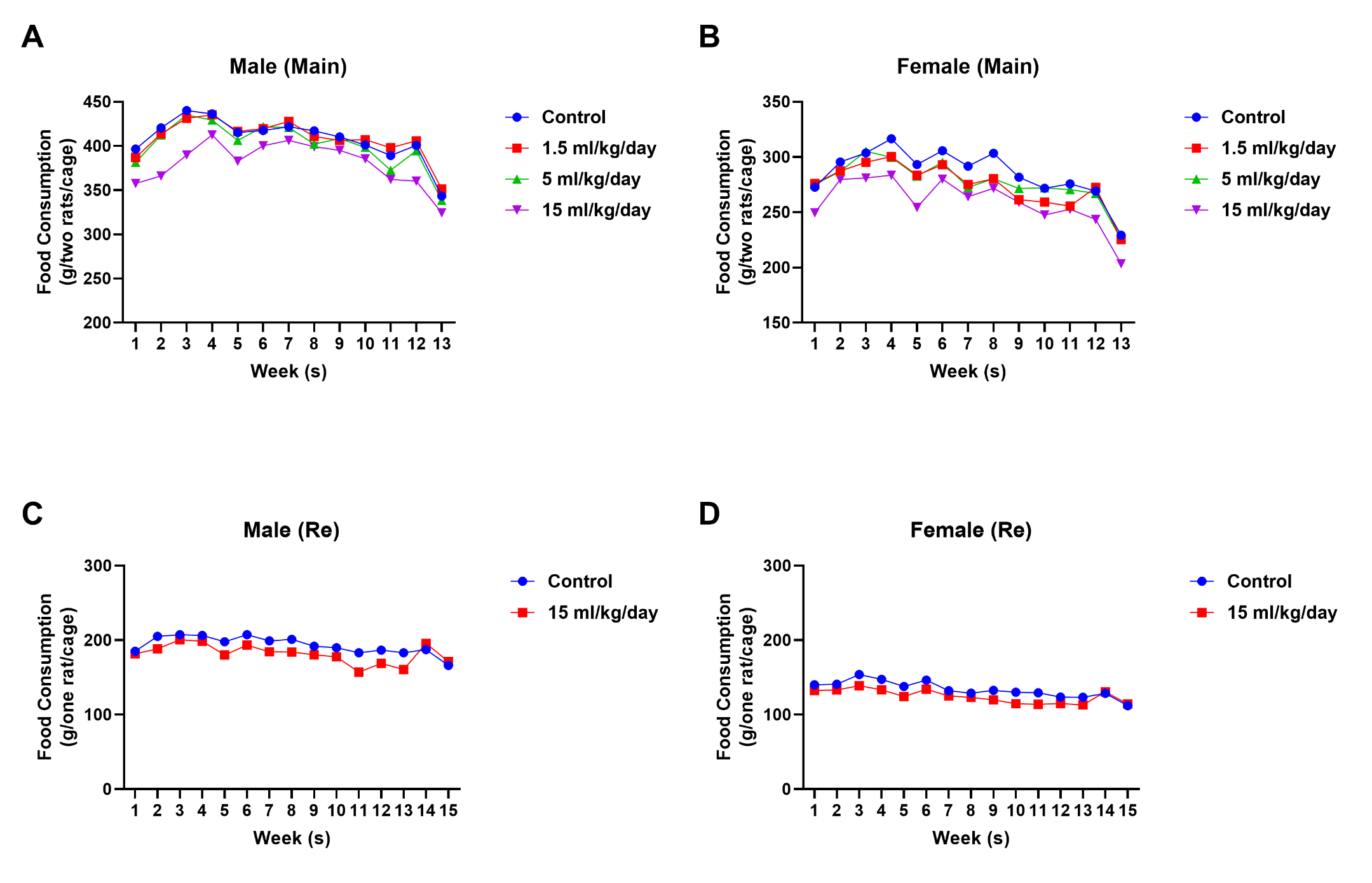
**

**Figure S2. Food consumption (g/rat(s)/cage) of 90-Day Subchronic Oral Toxicity Study**

(A) Male rats – Main study (n=10 for all groups); (B) Female rats – Main study (n=10 for all groups); (C) Male rats – Recovery study (n=5 for all groups); (D) Female rats – Recovery study (n=5 for all groups)

**
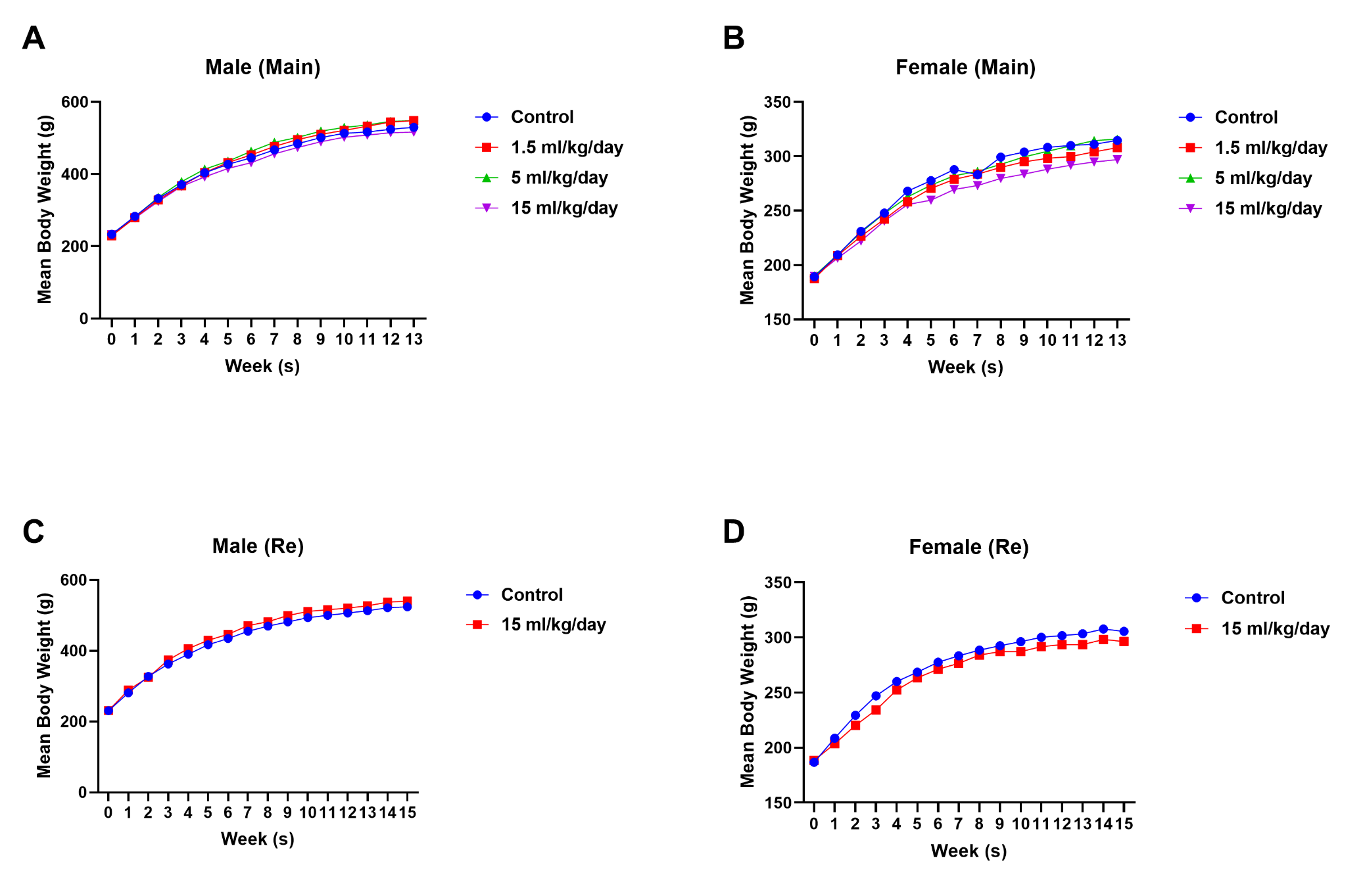
**

**Figure S3. The growth curve (mean body weight) of 90-Day Subchronic Oral Toxicity Study**

(A) Male rats – Main study (n=10 for all groups); (B) Female rats – Main study (n=10 for all groups); (C) Male rats – Recovery study (n=5 for all groups); (D) Female rats – Recovery study (n=5 for all groups)

| **Table S1. Acute Oral Toxicity Study of Symbiota^®^ (MS-20) in Rats — Mean Body Weight (N=6)** | | | | | | | |
| --- | --- | --- | --- | --- | --- | --- | --- |
|  |  |  |  |  |  |  |  |
| **Sex** | **Dose** | **Mean Body Weight (g) ± S.D.** | | | **Weight Gain (g) ± S.D. at Day 15** |  |  |
|  | **(ml/kg)** | **Day 1** | **Day 8** | **Day 15** |  |  |  |
| Male | 0 | 179.8 ± 5.2 | 278.7 ± 12.6 | 347.7 ± 17.1 | 167.8 ± 13.9 |  |  |
|  | 5 | 182.3 ± 7.4 | 283.7 ± 9.2 | 357.2 ± 16.2 | 174.8 ± 17.3 |  |  |
|  | 10 | 178.8 ± 4.3 | 281.5 ± 7.8 | 354.5 ± 11.0 | 175.7 ± 10.7 |  |  |
|  | 20 | 178.7 ± 3.3 | 283.0 ± 9.1 | 361.3 ± 10.4 | 182.7 ± 11.8 |  |  |
| Female | 0 | 139.3 ± 4.8 | 199.7 ± 13.7 | 226.8 ± 15.6 | 87.5 ± 12.3 |  |  |
|  | 5 | 139.2 ± 5.4 | 202.0 ± 14.3 | 231.0 ± 21.3 | 91.8 ± 18.9 |  |  |
|  | 10 | 139.7 ± 4.6 | 201.7 ± 11.7 | 233.7 ± 16.5 | 94.0 ± 15.3 |  |  |
|  | 20 | 139.0 ± 3.7 | 200.3 ± 10.3 | 228.5 ± 14.3 | 89.5 ± 15.7 |  |  |

| **Table S2. 28-Day Subacute Oral Toxicity Study of Symbiota^®^ (MS-20) in Rats - Mortality and Ophthalmologic Abnormality Examination** | | | | | | | | |
| --- | --- | --- | --- | --- | --- | --- | --- | --- |
|  |  |  |  |  |  |  |  |  |
| Dose (ml/kg/day) | 0 | | 1.5 | | 5 | | 15 | |
| Sex | M | F | M | F | M | F | M | F |
| Number of animals | 10 | 10 | 10 | 10 | 10 | 10 | 10 | 10 |
| Mortality | 0/10 | 0/10 | 0/10 | 0/10 | 0/10 | 0/10 | 0/10 | 0/10 |
| Ophtaalmologic abnormality |  |  |  |  |  |  |  |  |
| Before initiation (Day 0) | 0/10 | 0/10 | 0/10 | 0/10 | 0/10 | 0/10 | 0/10 | 0/10 |
| Before necropsy (Day 28) | 0/10 | 0/10 | 0/10 | 0/10 | 0/10 | 0/10 | 0/10 | 0/10 |
| N/N: Number of rats with death or abnormality/Number of rats examined | | | |  |  |  |  |  |
| Control: injection grade water (15 ml/kg/day) | |  |  |  |  |  |  |  |
| M:Male F:Female |  |  |  |  |  |  |  |  |

| **Table S3. 28-Day Subacute Oral Toxicity Study of Symbiota® (MS-20) in Rats - Clinical Signs and Total Incidence** | | | | | | | |
| --- | --- | --- | --- | --- | --- | --- | --- |
|  |  |  |  |  |  |  |  |
| Dose (ml/kg/day) | 0 | 1.5 | 5 | 15 |  |  |  |
| Number of animals | 10 | 10 | 10 | 10 |  |  |  |
| Male |  |  |  |  |  |  |  |
| Salivation | 0/10 | 0/10 | 0/10 | 9/10 |  |  |  |
| Hair loss | 0/10 | 0/10 | 0/10 | 1/10 |  |  |  |
| Wound | 0/10 | 0/10 | 0/10 | 1/10 |  |  |  |
| Female |  |  |  |  |  |  |  |
| Audible respiration | 0/10 | 0/10 | 0/10 | 1/10 |  |  |  |
| Salivation | 0/10 | 0/10 | 0/10 | 10/10 |  |  |  |
| N/N: Number of rats with clinical signs/Number of rats examined | | | |  |  |  |  |
| Control: injection grade water (15 ml/kg/day) | |  |  |  |  |  |  |

| **Table S4. 28-Day Subacute Oral Toxicity Study of Product Code MicrSoy-20 (MS-20) in Rats -Average Daily Food Consumption (Mean ± S.D., N=10)** | | | | | | |
| --- | --- | --- | --- | --- | --- | --- |
|  |  |  |  |  |  |  |
| **Sex** | **Dose** | **Average Daily Food Consumption (g/kg/day)** | | | |  |
|  | **(ml/kg)** | **Week 1** | **Week 2** | **Week 3** | **Week 4** |  |
| Male | 0 | 158.6 ± 8.4 | 132.6 ± 5.2 | 110.6 ± 6.8 | 96.6 ± 5.3 |  |
|  | 5 | 169.2 ±11.0 | 132.6 ± 5.2 | 110.0 ± 4.9 | 96.0 ± 3.6 |  |
|  | 10 | 162.6 ± 5.0 | 128.2 ± 5.5 | 105.4 ± 4.0 | 92.4 ± 3.4 |  |
|  | 20 | 153.2 ± 10.4 | 120.8 ± 3.8* | 100.4 ± 4.2* | 90.0 ± 3.l |  |
| Female | 0 | 154.8 ± 10.6 | 124.2 ± 4.6 | 115.8 ± 6.5 | 107.6 ± 6.8 |  |
|  | 5 | 158.8 ± 18.0 | 122.8 ± 4.4 | 115.2 ± 6.2 | 104.4 ± 2.4 |  |
|  | 10 | 155.2 ± 18.4 | 124.6±10.4 | 109.6 ± 9.4 | 100.4 ± 4.4 |  |
|  | 20 | 141.0 ± 9.8 | 109.8±11.6* | 103.4 ± 10.2 | 92.6 ± 8.2* |  |
| * Significant difference compared to the control group (p < 0.05) | | | | | | |

| **Table S5. 28-Day Subacute Oral Toxicity Study of Symbiota^®^ (MS-20) in Rats -Urinalyses (Mean ± S.D., N=10)** | | | | | | | |
| --- | --- | --- | --- | --- | --- | --- | --- |
|  |  |  |  |  |  |  |  |
| Sex | Parameter | Dose (ml/kg/day) | | | |  |  |
|  |  | 0 | 1.5 | 5 | 15 |  |  |
| Male | volume (ml) | 28.4 ± 5.9 | 24.4 ± 6.0 | 29.8 ± 8.7 | 31.2 ± 6.1 |  |  |
|  | specific gravity | 1.02 ± 0.006 | 1.022 ± 0.005 | 1.018 ± 0.005 | 1.017 ± 0.003 |  |  |
|  | pH | 7.02 ± 0.16 | 6.85 ± 0.47 | 6.95 ± 0.28 | 7.25 ± 0.54 |  |  |
|  | urobilinogen (EU/dl) | 0.20 ± 0.00 | 0.20 ± 0.00 | 0.20 ± 0.00 | 0.20 ± 0.00 |  |  |
| Female | volume (ml) | 14.7 ± 6.3 | 14.7 ± 3.7 | 14.3 ± 4.3 | 18.7 ± 5.4 |  |  |
|  | specific gravity | 1.025 ± 0.005^a^ | 1.024 ± 0.004 | 1.024 ± 0.006 | 1.020 ± 0.006 |  |  |
|  | pH | 6.60 ± 0.46 | 6.80 ± 0.26 | 6.90 ± 0.32 | 7.30 ± 0.26* |  |  |
|  | urobilinogen (EU/dl) | 0.20 ± 0.00 | 0.20 ± 0.00 | 0.20 ± 0.00 | 0.20 ± 0.00 |  |  |
| ^a^ n=9, one datum was out of the detection limit and was not included | | | |  |  |  |  |
| * Significant difference compared to the control group (p < 0.05) | | | | | | |  |

| **Table S6. 28-Day Subacute Oral Toxicity Study of Symbiota^®^(MS-20) in Rats-Hematology Parameters (Mean ± S.D, N=10)** | | | | | | | | | | | | | |  |  |  |
| --- | --- | --- | --- | --- | --- | --- | --- | --- | --- | --- | --- | --- | --- | --- | --- | --- |
|  | |  | | |  | |  |  | |  | | | |  |  |  |
| Parameter | Male (ml/kg/day) | | | | | | | | Female (ml/kg/day) | | | | | | | |
|  | 0 | | 1.5 | 5 | | 15 | | | 0 | | 1.5 | 5 | 15 | | | |
| Red blood cells (RBCs, xl0^6^ /µl) | 7.48 ± 0.43 | | 7.52 ± 0.24 | 7.58 ± 0.40 | | 7.88 ± 0.28 | | | 7.24 ± 0.25 | | 7.38 ± 0.33 | 7.32 ± 0.28 | 7.39 ± 0.38 | | | |
| Hemoglobin (Hb; g/dl) | 15.03 ± 0.49 | | 14.83 ± 0.40 | 14.92 ± 0.67 | | 15.18 ± 0.49 | | | 14.55 ± 0.32 | | 14.76 ± 0.52 | 14.57 ± 0.48 | 14.53 ± 0.62 | | | |
| Hematocrit (%) | 45.47 ± 1.50 | | 44.91 ± 1.23 | 45.84 ± 1.62 | | 46.94 ± 1.56 | | | 43.24 ± 1.05 | | 43.57 ± 1.72 | 43.63 ± 1.59 | 43.47 ± 2.00 | | | |
| Mean corpuscular volume (µm^3^ ) | 60.89 ± 2.02 | | 59.78 ± 1.91 | 60.53 ± 1.98 | | 59.57 ± 0.54 | | | 59.74 ± 1.25 | | 59.01 ± 1.08 | 59.59 ± 1.05 | 58.91 ± 1.85 | | | |
| Mean corpuscular Hb (pg) | 20.13 ± 0.88 | | 19.74 ± 0.64 | 19.71 ± 0.93 | | 19.27 ± 0.43 | | | 20.10 ± 0.57 | | 19.99 ± 0.52 | 19.89 ± 0.53 | 19.70 ± 0.79 | | | |
| Mean corpuscular Hb concentration (%) | 33.06 ± 0.51 | | 33.03 ± 0.45 | 32.54 ± 0.67 | | 32.33 ± 0.68* | | | 33.66 ± 0.50 | | 33.88 ± 0.70 | 33.40 ± 0.5 l | 33.44 ± 0.59 | | | |
| Platelets count (x10^3^ /µl) | 914.3 ± 54.0 | | 904.1 ± 49.7 | 919.4 ± 50.7 | | 1039.5 ± 112.2* | | | 906.8 ± 114.1 | | 930.7 ± 135.7 | 941.4 ± 110.1 | 1014.7 ± 65.l | | | |
| White blood cells (WBCs, xl0^3^ /µl) | 6.91 ± 1.32 | | 7.18 ± 1.71 | 7.02 ± 1.15 | | 9.30 ± 1.88* | | | 3.77 ± 0.72 | | 4.28 ± 0.59 | 3.75 ± 0.81 | 4.10 ± 0.99 | | | |
| Bands (%) | 0 | | 0 | 0 | | 0.1 ± 0.3 | | | 0.1 ± 0.3 | | 0 | 0 | 0 | | | |
| Lymphocytes (%) | 88.2 ± 4.7 | | 88.6 ± 6.2 | 88.9 ± 4.6 | | 88.2 ± 5.5 | | | 88.4 ± 7.0 | | 89.4 ± 5.6 | 90.2 ± 4.4 | 89.8 ± 6.3 | | | |
| Monocytes (%) | 3.2 ± 2.1 | | 2.5 ± 1.6 | 2.6 ± 2.3 | | 2.5 ± 1.3 | | | 2.0 ± 1.6 | | 2.0 ± 1.6 | 1.5 ± 1.4 | 2.1 ± 1.9 | | | |
| Neutrophils (%) | 8.2 ± 3.9 | | 8.7 ± 5.5 | 8.4 ± 3.2 | | 8.6 ± 5.l | | | 8.9 ± 5.4 | | 8.0 ± 5.5 | 7.6 ± 3.4 | 7.0 ± 4.7 | | | |
| Eosinophils (%) | 0.4 ± 0.5 | | 0.2 ± 0.4 | 0.1 ± 0.3 | | 0.6 ± 0.7 | | | 0.6 ± 0.8 | | 0.6±0.8 | 0.7 ± 0.8 | 1.1 ± 1.0 | | | |
| Basophils (%) | 0 | | 0 | 0 | | 0 | | | 0 | | 0 | 0 | 0 | | | |
| Granulocytes (%) | 8.6 ± 3.7 | | 8.9 ± 5.4 | 8.5 ± 3.1 | | 9.3 ± 5.4 | | | 9.6 ± 5.8 | | 8.6 ± 5.9 | 8.3 ± 3.9 | 8.1 ± 4.9 | | | |
| Prothrombin time (sec) | 15.8 ± 1.5 | | 16.1 ± 1.4 | 16.5 ± 1.3 | | 15.8 ± 1.3 | | | 15.0 ±1.0 | | 15.1 ± 0.8 | 14.9 ± 0.83^a^ | 15.5 ± 0.9 | | | |
| Activated partial thromboplastine time (sec) | 20.2±2.9 | | 21.1 ± 2.5 | 22.8 ± 2.5 | | 22.3 ± 2.1 | | | 16.1 ± 1.7 | | 16.3 ± 2.1 | 17.5 ± 2.03^a^ | 17.4 ± 0.9 | | | |
| ^a^ n=9, one sample was excluded due to blood coagulation | | |  |  | |  | | |  | |  |  |  | | | |
| * Significant difference compared to the control group (p < 0.05) | | | | | | | | | |  | | | |  |  |  |

| **Table S7. 28-Day Subacute Oral Toxicity Study of Symbiota^®^ (MS-20) in Rats - Serum chemistry Parameters (Mean ± S.D., N=10)** | | | | | | | | | | | | |  | |  |
| --- | --- | --- | --- | --- | --- | --- | --- | --- | --- | --- | --- | --- | --- | --- | --- |
|  |  | |  | |  | |  | |  | |  | |  | |  |
| Parameter | Male (ml/kg/day) | | | | | | | | Female (ml/kg/day) | | | | | | |
|  | 0 | 1.5 | | 5 | | 15 | | 0 | | 1.5 | | 5 | | 15 | |
| Alanine aminotransferase (U/l) | 29.9±4.6 | 30.0±3.4 | | 29.1±3.0 | | 25.3±4.4* | | 25.0±3.9 | | 24.5±4.2 | | 25.0±5.4 | | 23.0±4.5 | |
| Aspartate aminotransferase (U/1) | 170.6±25.6 | 151.8±35.5 | | 134.8±22.7* | | 132.9±40.5* | | 142.3±35.6 | | 119.8±24.7 | | 136.4±37.5 | | 121.2±27.2 | |
| Alkaline phosphatase (U/1) | 171.8±39.7 | 178.8±20.5 | | 166.3±29.6 | | 151.9±38.6 | | 99.7±21.8 | | 98.5±26.l | | 95.7±26.9 | | 94.5±22.3 | |
| Total protein (g/dl) | 4.74±0.16 | 4.83±0.20 | | 4.83±0.16 | | 4.96±0.26 | | 5.11 ±0.19 | | 5.28±0.36 | | 5.24±0.21 | | 5.12±0.37 | |
| Albumin (g/dl) | 3.l0±0.08 | 3.14±0.08 | | 3.13±0.11 | | 3.20±0.14 | | 3.38±0.14 | | 3.46±0.23 | | 3.43±0.08 | | 3.36±0.20 | |
| Albumin/Globulin ratio | 1.90±0.16 | 1.86±0.13 | | 1.85±0.ll | | 1.82±0.08 | | 1.93±0.13 | | 1.88±0.08 | | 1.90±0.18 | | 1.93±0.21 | |
| y-glutamyltransferase (U/1) | 0.60±0.38^a^ | 0.60±0.30^b^ | | 0.60±0.24 | | 0.68±0.41^a^ | | 0.65±0.35 | | 0.57±0.29 | | 0.65±0.30 | | 0.59±0.35^a^ | |
| Direct bilirubin (mg/dl) | 0.02±0.01 | 0.01 ±0.01 | | 0.02±0.01 | | 0.01±0.01 | | 0.01±0.00 | | 0.01±0.01^b^ | | 0.00±0.01^a^ | | 0.00±0.01 | |
| Total bilirubin (mg/dl) | 0.02±0.01^b^ | 0.03±0.02^a^ | | 0.02±0.01 | | 0.02±0.02^a^ | | 0.02±0.01 | | 0.01±0.02^a^ | | 0.02±0.02^d^ | | 0.01 ±0.01^c^ | |
| Triglycerides (mg/dl) | 56.6±22.7 | 54.9±19.4 | | 53.8±27.9 | | 57.0±21.4 | | 11.2±4.4 | | 17.5±7.4 | | 13.0±3.4 | | 18.1 ±7.6* | |
| Cholesterol (mg/dl) | 59.7±6.7 | 61.8±7.7 | | 64.5±7.3 | | 62.3±6.2 | | 79.1±8.8 | | 69.6±12.4 | | 80.4±11.5 | | 85.5±19.1 | |
| Lactate dehydrogenase (U/1) | 1916.3±455.0 | 1596.4±661.4 | | 1292.3±446.6 | | 1250.7±677.8* | | 1583.2±595.1 | | 1314.5±444.7 | | 1526.1±654.6 | | 1321.7±398.9 | |
| Creatine phosphate kinase (U/l) | 1381.5±466.2 | 1103.8±445.7 | | 855.5±289.2* | | 849.6±443.4* | | 1088.2±366.1 | | 887.3±370.2 | | 973.9±486.0 | | 812.6±263.9 | |
| Glucose (mg/dl) | 112.5±32.6 | 97.0±23.l | | 102.0±25.2 | | 89.7±14.2 | | 83.6±19.1 | | 101.0±23.8 | | 93.0±8.5 | | 88.6±15.7 | |
| Blood urea nitrogen (mg/dl) | 18.02±3.12 | 17.15±2.34 | | 17.04±2.59 | | 16.34±2.01 | | 23.05±4.27 | | 22.66±4.02 | | 23.51±2.62 | | 23.80±3.90 | |
| Uric acid (mg/dl) | 1.07±0.21 | 0.96±0.12 | | 0.96±0.07 | | 1.01±0.15 | | 1.16±0.32 | | l.00±0.17 | | 1.02±0.28 | | 0.98±0.21 | |
| Creatinine (mg/dl) | 0.70±0.07 | 0.73±0.07 | | 0.69±0.07 | | 0.70±0.09 | | 0.80±0.01 | | 0.79±0.07 | | 0.82±0.06 | | 0.79±0.09 | |
| Calcium (mg/dl) | 10.27±0.36 | 10.18±0.17 | | 10.25±0.17 | | 10.36±0.34 | | 10.05±0.29 | | 10.28±0.29 | | 10.22±0.30 | | 10.30±0.40 | |
| Phosphorus (mg/dl) | 8.64±0.59 | 8.85±0.56 | | 8.84±0.60 | | 9.06±0.57 | | 7.11±0.56 | | 7.02±0.85 | | 7.40±1.08 | | 7.25±0.61 | |
| Sodium (mEq/1) | 146.2±2.0 | 146.3±0.8 | | 147.2±1.3 | | 147.l ± 1.3 | | 146.1±1.6 | | 146.3±1.3 | | 147.1±1.7 | | 147.3±1.2 | |
| Potassium (mEq/1) | 4.18±0.19 | 4.11±0.34 | | 4.01±0.34 | | 4.32±0.39 | | 3.77±0.31 | | 3.80±0.37 | | 3.76±0.29 | | 4.04±0.40 | |
| Chloride (mEq/1) | 100.9±2.8 | 101.6± 1.3 | | 103.2±2.2* | | 103.6±1.6* | | 106.1±1.8 | | 105.6±1.0 | | 106.7±1.8 | | 107.5±2.1 | |
| a: n=9, one datum was out of the detection limit and was not calculated. | | | | |  | |  | |  | |  | |  | |  |
| b: n=8, two data were out of the detection limit and were not calculated. | | | | |  | |  | |  | |  | |  | |  |
| c: n=7, three data were out of the detection limit and were not calculated. | | | | |  | |  | |  | |  | |  | |  |
| d: n=6, four data were out of the detection limit and were not calculated. | | | | |  | |  | |  | |  | |  | |  |
| * Significant difference compared to the control group (p < 0.05) | | | | | | |  | |  | |  | |  | |  |

| **Table S8. 28-Day Subacute Oral Toxicity Study of Symbiota^®^ (MS-20) in Male Rats - Organ Weights and Organ to Brain Weight Ratio (Mean ± S.D., N=10)** | | | | | | | | | |
| --- | --- | --- | --- | --- | --- | --- | --- | --- | --- |
|  |  |  |  |  |  |  |  |  |  |
| Parameter | Male (ml/kg/day) | | | | Female (ml/kg/day) | | | |  |
|  | 0 | 1.5 | 5 | 15 | 0 | 1.5 | 5 | 15 |  |
| Brain (g) | 2.01±0.08 | 2.01±0.11 | 2.02±0.07 | 2.05±0.06 | l.85±0.07 | l.89±0.07 | 1.87±0.05 | 1.83±0.06 |  |
| Liver (g) | 14.56±0.41 | 14.28±1.61 | 13.96±1.24 | 14.15±1.82 | 8.76±0.63 | 8.75±0.79 | 8.85±0.67 | 8.90±1.66 |  |
| Ratio | 7.24±0.32 | 7.12±0.80 | 6.92±0.57 | 6.93±1.02 | 4.73±0.35 | 4.65±0.47 | 4.75±0.39 | 4.88±1.0l |  |
| Kidneys (g) | 3.28±0.28 | 3.23±0.32 | 3.29±0.34 | 3.29±0.26 | l.98±0.17 | 2.03±0.23 | l.93±0.19 | I.92±0.27 |  |
| Ratio | 1.63±0.12 | 1.61±0.17 | 1.63±0.14 | 1.61±0.15 | 1.07±0.09 | 1.08±0.12 | 1.03±0.10 | 1.05±0.18 |  |
| Spleen (g) | 0.93±0.08 | 0.98±0.12 | 0.93±0.14 | 0.89±0.09 | 0.63±0.12 | 0.61±0.08 | 0.55±0.11 | 0.57±0. l 1 |  |
| Ratio | 0.47±0.06 | 0.49±0.06 | 0.46±0.06 | 0.44±0.05 | 0.34±0.06 | 0.32±0.04 | 0.30±0.06 | 0.31±0.06 |  |
| Heart (g) | 1.37±0.13 | 1.37±0.13 | 1.32±0.12 | 1.34±0.12 | 0.96±0.07 | 0.90±0.04 | 0.95±0.11 | 0.88±0.11 |  |
| Ratio | 0.68±0.08 | 0.68±0.06 | 0.66±0.05 | 0.66±0.07 | 0.52±0.04 | 0.48±0.03 | 0.51±0.06 | 0.48±0.07 |  |
| Adrenals (g) | 0.062±0.011 | 0.067±0.011 | 0.067±0.013 | 0.061±0.009 | 0.075±0.007 | 0.075±0.013 | 0.075±0.013 | 0.070±0.018 |  |
| Ratio^a^ (%) | 3.09±0.59 | 3.34±0.61 | 3.30±0.66 | 2.99±0.37 | 4.07±0.43 | 4.00±0.67 | 3.99±0.66 | 3.84±0.97 |  |
| Thymus (g) | 0.679±0.087 | 0.625±0.118 | 0.602±0.136 | 0.671±0.103 | 0.522±0.082 | 0.504±0.075 | 0.487±0.067 | 0.470±0.l 10 |  |
| Ratio | 0.34±0.05 | 0.31±0.06 | 0.30±0.06 | 0.33±0.05 | 0.28±0.04 | 0.27±0.05 | 0.26±0.04 | 0.26±0.06 |  |
| Testes (g) | 3.53±0.19 | 3.71±0.20 | 3.75±0.30 | 3.75±0.20 | n/a | n/a | n/a | n/a |  |
| Ratio | 1.76±0.11 | 1.85±0.12 | 1.85±0.11 | 1.83±0.10 | n/a | n/a | n/a | n/a |  |
| Ratio: organ weight/brain weight | |  |  |  |  |  |  |  |  |
| a: (organ weight/brain weight) x 100 | |  |  |  |  |  |  |  |  |
| n/a: not applicable |  |  |  |  |  |  |  |  |  |

| **Table S9. 28-Day Subacute Oral Toxicity Study of Symbiota^®^ (MS-20) in Rats - Gross Necropsy Findings (N/N)** | | | | | | |
| --- | --- | --- | --- | --- | --- | --- |
|  |  |  |  |  |  |  |
| Dose (ml/kg/day) | 0 | 1.5 | 5 | 15 |  |  |
| Number of animals | 10 | 10 | 10 | 10 |  |  |
| Male |  |  |  |  |  |  |
| Urinary bladder |  |  |  |  |  |  |
| Fat-like mass, serosal surface | 1/10 | 0/10 | 0/10 | 0/10 |  |  |
| Spleen |  |  |  |  |  |  |
| White spots | 0/10 | 0/10 | 1/10 | 0/10 |  |  |
| Thymus |  |  |  |  |  |  |
| Hemorrhage, anterior portion | 0/10 | 0/10 | 1/10 | 0/10 |  |  |
| Female |  |  |  |  |  |  |
|  |  |  |  |  |  |  |
| Audible respiration | 1/10 | 1/10 | 0/10 | 0/10 |  |  |
| Salivation |  |  |  |  |  |  |
| N/N: Number of rats with clinical signs/Number of rats examined | | |  |  |  |  |

| **Table S10. 28-Day Subacute Oral Toxicity Study of Symbiota^®^ (MS-20) in Male Rats - Incidence and Severity of Histopathology Lesions (N/N)** | | | | | | | |
| --- | --- | --- | --- | --- | --- | --- | --- |
|  |  |  |  |  |  |  |  |
| Sex | Male | | Female | |  |  |  |
| Dose (ml/kg/day) | 0 | 15 | 0 | 15 |  |  |  |
| Number of animals | 10 | 10 | 10 | 10 |  |  |  |
| Kidney |  |  |  |  |  |  |  |
| Mineralization, renal tubules |  |  |  |  |  |  |  |
| Incidence | 1/10 | 0/10 | 3/10 | 0/10 |  |  |  |
| Degree of severity^a^ |  |  |  |  |  |  |  |
| Minimal | 1/10 | 0/10 | 3/10 | 3/10 |  |  |  |
| Slight | 1/10 | 0/10 | 0/10 | 2/10 |  |  |  |
| Nephropathy, progressive, chronic | |  |  |  |  |  |  |
| Incidence | 2/10 | 0/10 | 0/10 | 0/10 |  |  |  |
| Degree of severity^a^ |  |  |  |  |  |  |  |
| Minimal | 2/10 | 0/10 | 0/10 | 0/10 |  |  |  |
|  |  |  |  |  |  |  |  |
| Prostate |  |  |  |  |  |  |  |
| Infiltration, lymphocytic cells |  |  |  |  |  |  |  |
| Incidence | 0/10 | 2/10 |  |  |  |  |  |
| Degree of severity^a^ |  |  |  |  |  |  |  |
| Minimal | 0/10 | 2/10 |  |  |  |  |  |
| N/N: Number of rats with microscopic lesions/Number of rats examined | | | |  |  |  |  |
| a: Degrees of lesions were graded from one to five depending on severity (EPL) | | | | |  |  |  |

| **Table S11. 90-Day Subchronic Oral Toxicity Study Organ - Incidence of Clinical Observation** | | | | | | | | | |
| --- | --- | --- | --- | --- | --- | --- | --- | --- | --- |
|  | | | | | | | | | |
| **Main study** | | | | | | | | | |
| Group† | C | | S-L | | S-M | | S-H | | |
| Dose (mL/kg) | 0 | | 1.5 | | 5 | | 15 | | |
| Sex | Male | Female | Male | Female | Male | Female | Male | Female |  |
| Abnormality | 0/10 | 0/10 | 0/10 | 0/10 | 0/10 | 0/10 | 0/10 | 0/10 |  |
|  |  |  |  |  |  |  |  |  |  |
| **Recovery study** | | | | | | | | | |
| Group† | Re-C | | | | Re-H | | | | |
| Dose (mL/kg) | 0 | | | | 15 | | | | |
| Sex | Male | | Female | | Male | | Female | | |
| Abnormality | 0/5 | | 0/5 | | 0/5 | | 0/5 | | |
| n/n: Number of rats with abnormalities/Number of rats in group. †C: control; S-L: Symbiota® low dose group; S-M: Symbiota® middle dose group; S-H: Symbiota® high dose group; Re-C: recovery control group; Re-H: recovery high dose group. | | | | | | | | | |

| **Table S12. 90-Day Subchronic Oral Toxicity Study Organ - Incidence of Mortality** | | | | | | | | | |
| --- | --- | --- | --- | --- | --- | --- | --- | --- | --- |
|  | | | | | | | | | |
| **Main study** | | | | | | | | | |
| Group† | C | | S-L | | S-M | | S-H | | |
| Dose (mL/kg) | 0 | | 1.5 | | 5 | | 15 | | |
| Sex | Male | Female | Male | Female | Male | Female | Male | Female |  |
| Abnormality | 0/10 | 0/10 | 0/10 | 0/10 | 0/10 | 0/10 | 0/10 | 0/10 |  |
|  |  |  |  |  |  |  |  |  |  |
| **Recovery study** | | | | | | | | | |
| Group† | Re-C | | | | Re-H | | | | |
| Dose (mL/kg) | 0 | | | | 15 | | | | |
| Sex | Male | | Female | | Male | | Female | | |
| Abnormality | 0/5 | | 0/5 | | 0/5 | | 0/5 | | |
| n/n: Number of rats with abnormalities/Number of rats in group. †C: control; S-L: Symbiota® low dose group; S-M: Symbiota® middle dose group; S-H: Symbiota® high dose group; Re-C: recovery control group; Re-H: recovery high dose group. | | | | | | | | | |

| **Table S13. 90-Day Subchronic Oral Toxicity Study Organ - Incidence of Gross Finding** | | | | | | | | | |
| --- | --- | --- | --- | --- | --- | --- | --- | --- | --- |
|  | | | | | | | | | |
| **Main study** | | | | | | | | | |
| Group† | C | | S-L | | S-M | | S-H | | |
| Dose (mL/kg) | 0 | | 1.5 | | 5 | | 15 | | |
| Sex | Male | Female | Male | Female | Male | Female | Male | Female |  |
| Abnormality | 0/10 | 0/10 | 0/10 | 0/10 | 0/10 | 0/10 | 0/10 | 0/10 |  |
|  |  |  |  |  |  |  |  |  |  |
| **Recovery study** | | | | | | | | | |
| Group† | Re-C | | | | Re-H | | | | |
| Dose (mL/kg) | 0 | | | | 15 | | | | |
| Sex | Male | | Female | | Male | | Female | | |
| Abnormality | 0/5 | | 0/5 | | 0/5 | | 0/5 | | |
| n/n: Number of rats with abnormalities/Number of rats in group. †C: control; S-L: Symbiota® low dose group; S-M: Symbiota® middle dose group; S-H: Symbiota® high dose group; Re-C: recovery control group; Re-H: recovery high dose group. | | | | | | | | | |

| **Table S14. 90-Day Subchronic Oral Toxicity Study Organ - Food Consumption** | | | | | | | | |
| --- | --- | --- | --- | --- | --- | --- | --- | --- |
|  | | | | | | | | |
| **Main study** | | | | | | | | |
| Group† | C | | S-L | | S-M | | S-H | |
| Dose (mL/kg) | 0 | | 1.5 | | 5 | | 15 | |
| Sex | Male | Female | Male | Female | Male | Female | Male | Female |
| Week 1 | 396.6 ± 29.9 | 272.8 ± 10.8 | 387.0 ± 31.0 | 276.0 ± 19.2 | 381.6 ± 19.0 | 275.0 ± 22.3 | 357.6 ± 42.6 | 249.6 ± 8.1 |
| Week 2 | 420.6 ± 24.6 | 295.6 ± 10.1 | 413.8 ± 29.7 | 287.4 ± 16.0 | 412.4 ± 29.4 | 287.0 ± 15.3 | 366.2 ± 51.9 | 279.8 ± 9.9 |
| Week 3 | 440.2 ± 33.2 | 303.8 ± 14.0 | 431.4 ± 27.2 | 295.2 ± 19.1 | 435.0 ± 27.1 | 305.2 ± 23.2 | 389.8 ± 67.9 | 281.0 ± 13.5 |
| Week 4 | 436.4 ± 18.4 | 316.6 ± 23.8 | 435.4 ± 23.2 | 300.4 ± 13.8 | 429.2 ± 26.6 | 299.8 ± 30.7 | 412.6 ± 40.3 | 283.6 ± 17.2 |
| Week 5 | 415.4± 17.8 | 293.2 ± 23.9 | 416.6 ± 22.6 | 283.6 ± 25.9 | 406.4 ± 25.5 | 282.8 ± 16.5 | 382.8 ± 28.1 | 254.4 ± 14.7 |
| Week 6 | 417.6± 16.0 | 305.8 ± 18.0 | 419.6 ± 14.7 | 293.0 ± 13.1 | 421.8 ± 27.6 | 295.2 ± 23.9 | 400.4 ± 7.10 | 280.0 ± 6.00 |
| Week 7 | 421.8 ± 20.2 | 291.8 ± 16.5 | 427.8 ± 17.9 | 275.2 ± 9.40 | 421.0 ± 34.9 | 272.6 ± 22.3 | 406.4 ± 15.6 | 264.0 ± 15.3 |
| Week 8 | 417.2 ± 18.5 | 303.4 ± 10.3 | 410.8 ± 13.4 | 280.4 ± 10.7 | 402.2 ± 31.7 | 280.4 ± 22.9 | 399.2 ± 23.0 | 271.8 ± 20.3 |
| Week 9 | 410.4 ± 15.8 | 281.8 ± 16.4 | 406.4 ± 10.9 | 261.4 ± 9.80 | 408.4 ± 32.8 | 271.6 ± 21.1 | 395.0 ± 17.7 | 259.0 ± 7.70 |
| Week 10 | 401.2 ± 14.0 | 271.8 ± 13.2 | 407.2 ± 16.0 | 259.2 ± 7.90 | 398.4 ± 27.3 | 272.0 ± 21.0 | 385.6 ± 12.8 | 247.6 ± 15.0 |
| Week 11 | 389.2 ± 15.7 | 275.8 ± 14.4 | 398.0 ± 9.10 | 255.6 ± 17.0 | 372.6 ± 30.0 | 270.6 ± 16.8 | 362.2 ± 20.1 | 252.8 ± 4.10 |
| Week 12 | 400.8 ± 19.9 | 269.2 ± 11.8 | 406.0 ± 9.60 | 272.6 ± 27.0 | 394.6 ± 33.7 | 267.0 ± 25.7 | 360.4 ± 29.0 | 243.6 ± 13.6 |
| Week 13 | 343.4 ± 17.1 | 229.2 ± 9.60 | 351.4 ± 11.1 | 225.4 ± 9.00 | 338.4 ± 26.5 | 225.2 ± 15.9 | 324.4 ± 21.3 | 203.4 ± 22.4 |
|  |  |  |  |  |  |  |  |  |
| **Recovery study** | | | | | | | | |
| Group† | Re-C | | | | Re-H | | | |
| Dose (mL/kg) | 0 | | | | 15 | | | |
| Sex | Male | | Female | | Male | | Female | |
| Week 1 | 185.2 ± 11.5 | | 140.0 ± 11.1 | | 181.6 ± 19.7 | | 132.4 ± 9.40 | |
| Week 2 | 205.2 ± 16.6 | | 140.8 ± 15.3 | | 188.4 ± 25.7 | | 133.2 ± 8.50 | |
| Week 3 | 207.4 ± 21.3 | | 153.8 ± 15.3 | | 200.6 ± 24.3 | | 138.8 ± 5.90 | |
| Week 4 | 206.4 ± 16.3 | | 147.4 ± 14.8 | | 198.6 ± 25.0 | | 133.4 ± 6.40 | |
| Week 5 | 197.8 ± 11.8 | | 137.8 ± 13.9 | | 180.0 ± 22.2 | | 124.4 ± 2.10 | |
| Week 6 | 207.4 ± 10.4 | | 146.4 ± 10.7 | | 193.4 ± 24.7 | | 134.2 ± 7.70 | |
| Week 7 | 199.0 ± 12.6 | | 132.0 ± 14.0 | | 184.4 ± 26.3 | | 125.0 ± 4.90 | |
| Week 8 | 201.0 ± 14.2 | | 128.8 ± 11.2 | | 184.0 ± 24.0 | | 123.2 ± 3.10 | |
| Week 9 | 191.8 ± 11.6 | | 132.6 ± 13.4 | | 180.4 ± 27.2 | | 119.8 ± 5.60 | |
| Week 10 | 189.8 ± 14.5 | | 130.2 ± 14.2 | | 177.6 ± 24.1 | | 114.6 ± 7.50 | |
| Week 11 | 183.0 ± 17.0 | | 129.4 ± 11.1 | | 157.2 ± 26 1 | | 113.8 ± 11.3 | |
| Week 12 | 186.6 ± 12.9 | | 123.6 ± 6.00 | | 168.8 ± 19.4 | | 114.8 ± 6.70 | |
| Week 13 | 183.0 ± 15.2 | | 123.4 ± 6.60 | | 160.6 ± 23.8 | | 113.0 ± 8.20 | |
| Week 14 | 187.4 ± 16.3 | | 128.6 ± 9.30 | | 195.6 ± 24.5 | | 130.6 ± 11.4 | |
| Week 15 | 166.0 ± 14.7 | | 112.2 ± 10.5 | | 171.4 ± 20.6 | | 114.4 ± 8.30 | |
| Data were presented as mean ± S.D. n = 5 (n, sample size, two rats per cage) in main study; 5 animals per group in recovery study. † C: control; S-L: Symbiota® low dose group; S-M: Symbiota® middle dose group; S-H: Symbiota® high dose group; Re-C: recovery control group; Re-H: recovery high dose group. | | | | | | | | |

| **Table S15. 90-Day Subchronic Oral Toxicity Study Organ - Body weights** | | | | | | | | | |
| --- | --- | --- | --- | --- | --- | --- | --- | --- | --- |
|  | | | | | | | | | |
| **Main study** | | | | | | | | | |
| Group† | C | | S-L | | S-M | | S-H | |  |
| Dose (mL/kg) | 0 | | 1.5 | | 5 | | 15 | |  |
| Sex | Male | Female | Male | Female | Male | Female | Male | Female |  |
| Week 0^‡^ | 233.2 ± 8.70 | 189.2 ± 5.20 | 229.4 ± 9.50 | 187.6 ± 6.40 | 233.8 ± 8.50 | 190.0 ± 5.30 | 232.0 ± 9.60 | 189.5 ± 4.90 |  |
| Week 1 | 283.2 ± 9.90 | 209.4 ± 5.70 | 279.1 ± 17.8 | 208.8 ± 8.40 | 281.4 ± 11.6 | 209.5 ± 7.30 | 278.0 ± 16.0 | 206.4 ± 10.3 |  |
| Week 2 | 332.8 ± 14.7 | 231.0 ± 7.80 | 328.1 ± 23.9 | 226.6 ± 9.60 | 335.6 ± 15.9 | 230.1 ± 8.80 | 324.1 ± 21.3 | 222.2 ± 12.7 |  |
| Week 3 | 370.8 ± 19.0 | 247.9 ± 9.00 | 367.6 ± 29.8 | 242.2 ± 12.3 | 379.4 ± 18.6 | 247.6 ± 11.3 | 366.3 ± 27.9 | 240.5 ± 15.5 |  |
| Week 4 | 403.7 ± 20.5 | 268.0 ± 8.10 | 404.2 ± 35.1 | 258.2 ± 12.9 | 413.9 ± 20.2 | 262.7 ± 11.9 | 392.2 ± 35.2 | 255.8 ± 15.9 |  |
| Week 5 | 427.4 ± 21.7 | 277.8 ± 11.0 | 432.2 ± 39.9 | 270.6 ± 15.0 | 435.7 ± 20.0 | 273.6 ± 13.0 | 415.9 ± 37.9 | 259.8 ± 20.8 |  |
| Week 6 | 445.2 ± 23.3 | 287.9 ± 8.30 | 453.3 ± 42.2 | 278.9 ± 14.4 | 463.0 ± 23.9 | 282.3 ± 14.9 | 431.8 ± 38.3 | 269.6 ± 21.7 |  |
| Week 7 | 467.3 ± 22.2 | 283.3 ± 10.7 | 476.9 ± 45.0 | 283.7 ± 14.2 | 488.0 ± 27.2 | 286.2 ± 14.9 | 456.0 ± 43.6 | 273.2 ± 21.5 |  |
| Week 8 | 484.3 ± 21.8 | 299.4 ± 9.10 | 495.6 ± 48.0 | 289.9 ± 14.2 | 501.6 ± 31.4 | 292.8 ± 14.9 | 473.5 ± 47.1 | 279.8 ± 20.9 |  |
| Week 9 | 501.3 ± 22.8 | 304.0 ± 10.9 | 510.2 ± 50.2 | 295.1 ± 13.4 | 519.4 ± 33.9 | 299.6 ± 16.8 | 489.7 ± 47.2 | 283.8 ± 22.8 |  |
| Week 10 | 513.0 ± 20.9 | 308.4 ± 12.3 | 521.4 ± 51.3 | 298.3 ± 14.6 | 529.4 ± 32.9 | 304.8 ± 19.2 | 502.0 ± 49.1 | 288.2 ± 21.0 |  |
| Week 11 | 516.6 ± 21.4 | 310.1 ± 11.5 | 532.6 ± 52.4 | 299.9 ± 15.4 | 536.3 ± 33.5 | 309.7 ± 19.1 | 508.1 ± 51.2 | 291.9 ± 23.1 |  |
| Week 12 | 524.2 ± 21.9 | 311.2 ± 12.6 | 544.1 ± 52.8 | 304.3 ± 15.3 | 546.1 ± 36.6 | 314.6 ± 19.6 | 514.9 ± 52.9 | 294.9 ± 22.0 |  |
| Week 13 | 529.6 ± 17.6 | 314.7 ± 13.0 | 548.1 ± 56.5 | 308.0 ± 16.6 | 548.0 ± 35.2 | 315.9 ± 18.6 | 516.2 ± 49.9 | 297.1 ± 22.6 |  |
|  |  |  |  |  |  |  |  |  |  |
| **Recovery study** | | | | | | | | | |
| Group† | Re-C | | | | Re-H | | | | |
| Dose (mL/kg) | 0 | | | | 15 | | | | |
| Sex | Male | | Female | | Male | | Female | |  |
| Week 0^‡^ | 230.6 ± 8.30 | | 186.8 ± 8.80 | | 231.0 ± 5.90 | | 188.4 ± 5.80 | |  |
| Week 1 | 281.0 ± 10.2 | | 208.6 ± 8.80 | | 289.0 ± 8.40 | | 203.6 ± 10.9 | |  |
| Week 2 | 327.6 ± 14.2 | | 229.4 ± 10.8 | | 325.0 ± 32.9 | | 220.2 ± 12.6 | |  |
| Week 3 | 362.8 ± 18.0 | | 247.0 ± 12.6 | | 374.0 ± 36.5 | | 234.2 ± 17.6 | |  |
| Week 4 | 390.4 ± 19.3 | | 260.0 ± 16.7 | | 406.4 ± 43.1 | | 252.4 ± 11.9 | |  |
| Week 5 | 417.0 ± 20.2 | | 268.6 ± 14.1 | | 429.6 ± 48.7 | | 263.4 ± 13.6 | |  |
| Week 6 | 434.6 ± 22.5 | | 277.6 ± 14.9 | | 446.6 ±  53.9 | | 271.2 ± 14.9 | |  |
| Week 7 | 455.2 ± 24.4 | | 283.4 ± 17.3 | | 471.4 ± 57.8 | | 276.6 ± 12.4 | |  |
| Week 8 | 469.8 ± 27.7 | | 288.6 ± 19.2 | | 482.0 ± 63.8 | | 284.0 ± 9.40 | |  |
| Week 9 | 481.6 ± 28.8 | | 292.6 ± 16.3 | | 499.6 ± 63.1 | | 287.2 ± 14.4 | |  |
| Week 10 | 493.6 ± 29.0 | | 296.2 ± 17.2 | | 511.0 ± 63.3 | | 287.2 ± 12.4 | |  |
| Week 11 | 500.0 ± 30.1 | | 300.2 ± 18.1 | | 516.4 ± 66.2 | | 291.8 ± 9.20 | |  |
| Week 12 | 506.8 ± 31.1 | | 301.8 ± 17.4 | | 520.8 ± 67.3 | | 293.6 ± 7.60 | |  |
| Week 13 | 513.0 ± 31.2 | | 303.4 ± 15.6 | | 527.4 ± 70.8 | | 293.6 ± 1.50 | |  |
| Week 14 | 521.6 ± 30.7 | | 307.8 ± 20.5 | | 537.2 ± 68.4 | | 298.2 ± 7.20 | |  |
| Week 15 | 524.2 ± 33.8 | | 305.6 ± 19.8 | | 540.6 ± 69.5 | | 296.4 ± 6.60 | |  |
| Data were presented as mean ± S.D. of 10 animals per group in main study; 5 animals per group in recovery study. † C: control; S-L: Symbiota® low dose group; S-M: Symbiota® middle dose group; S-H: Symbiota® high dose group; Re-C: recovery control group; Re-H: recovery high dose group. ^‡^ Week 0: the day prior to administration of the test article or sterile water (control). | | | | | | | | | |

| **Table S16. 90-Day Subchronic Oral Toxicity Study Organ - Blood Coagulation Analysis** | | | | | | | | |
| --- | --- | --- | --- | --- | --- | --- | --- | --- |
|  | | | | | | | | |
| **Main study** | | | | | | | | |
| Group† | C | | S-L | | S-M | | S-H | |
| Dose (mL/kg) | 0 | | 1.5 | | 5 | | 15 | |
| Sex | Male | Female | Male | Female | Male | Female | Male | Female |
| Test parameters^#^ | | | | | | | | |
| PT (sec) | 12.96 ± 1.69 | 9.12 ± 0.20 | 13.63 ± 1.36 | 8.98 ± 0.22 | 13.70 ± 2.33 | 9.21 ± 0.21 | 11.29 ± 1.03 | 9.17 ± 0.23 |
| APTT (sec) | 18.79 ± 2.15 | 15.38 ± 1.05 | 18.52 ± 2.33 | 14.96 ± 1.03 | 18.93 ± 1.43 | 16.58 ± 1.23* | 18.40 ± 0.99 | 15.34 ± 0.94 |
|  |  |  |  |  |  |  |  |  |
| **Recovery study** | | | | | | | | |
| Group† | Re-C | | | | Re-H | | | |
| Dose (mL/kg) | 0 | | | | 15 | | | |
| Sex | Male | | Female | | Male | | Female | |
| Test parameters^#^ | | | | | | | | |
| PT (sec) | 13.64 ± 2.02 | | 9.00 ± 0.14 | | 14.24 ± 2.32 | | 9.16 ± 0.11 | |
| APTT (sec) | 18.92 ± 1.50 | | 16.60 ± 0.62 | | 18.54 ± 1.69 | | 16.56 ± 0.59 | |
| Data were presented as mean ± S.D. of 10 animals per group in main study; 5 animals per group in recovery study. * Significant difference compared to the control group (p < 0.05). ^#^PT: Prothrombin time; APTT: Activated partial thromboplastin time. †C: control; S-L: Symbiota® low dose group; S-M: Symbiota® middle dose group; S-H: Symbiota® high dose group; Re-C: recovery control group; Re-H: recovery high dose group. | | | | | | | | |

| **Table S17 90-Day Subchronic Oral Toxicity Study Organ - Urinalysis**  **Male Rats** | | | | | | | |  | **Female Rats** | | | | | | | |
| --- | --- | --- | --- | --- | --- | --- | --- | --- | --- | --- | --- | --- | --- | --- | --- | --- |
| Group† |  | C | S-L | S-M | S-H | Re-C | Re-H |  | Group† |  | C | S-L | S-M | S-H | Re-C | Re-H |
| Dose (mL/kg) |  | 0 | 1.5 | 5 | 15 | 0 | 15 |  | Dose (mL/kg) |  | 0 | 1.5 | 5 | 15 | 0 | 15 |
| Appearance | LT. YELLOW | 0/10 | 0/10 | 0/10 | 1/10 | 0/5 | 0/5 |  | Appearance | LT. YELLOW | 1/10 | 1/10 | 3/10 | 1/10 | 0/5 | 0/5 |
|  | YELLOW | 4/10 | 8/10 | 8/10 | 8/10 | 5/5 | 3/5 |  |  | YELLOW | 9/10 | 7/10 | 7/10 | 8/10 | 5/5 | 5/5 |
|  | DK. YELLOW | 6/10 | 2/10 | 2/10 | 1/10 | 0/5 | 2/5 |  |  | DK. YELLOW | 0/10 | 2/10 | 0/10 | 1/10 | 0/5 | 0/5 |
|  | LT. BROWN | 0/10 | 0/10 | 0/10 | 0/10 | 0/5 | 0/5 |  |  | LT. BROWN | 0/10 | 0/10 | 0/10 | 0/10 | 0/5 | 0/5 |
|  | BROWN | 0/10 | 0/10 | 0/10 | 0/10 | 0/5 | 0/5 |  |  | BROWN | 0/10 | 0/10 | 0/10 | 0/10 | 0/5 | 0/5 |
|  | DK. BROWN | 0/10 | 0/10 | 0/10 | 0/10 | 0/5 | 0/5 |  |  | DK. BROWN | 0/10 | 0/10 | 0/10 | 0/10 | 0/5 | 0/5 |
|  | ORANGE | 0/10 | 0/10 | 0/10 | 0/10 | 0/5 | 0/5 |  |  | ORANGE | 0/10 | 0/10 | 0/10 | 0/10 | 0/5 | 0/5 |
|  | DK. ORANGE | 0/10 | 0/10 | 0/10 | 0/10 | 0/5 | 0/5 |  |  | DK. ORANGE | 0/10 | 0/10 | 0/10 | 0/10 | 0/5 | 0/5 |
| Glucose | - | 10/10 | 10/10 | 10/10 | 10/10 | 5/5 | 5/5 |  | Glucose | - | 10/10 | 10/10 | 10/10 | 10/10 | 5/5 | 5/5 |
| Bilirubin | - | 5/10 | 4/10 | 8/10 | 6/10 | 2/5 | 4/5 |  | Bilirubin | - | 9/10 | 6/10 | 3/10 | 6/10 | 2/5 | 4/5 |
|  | +/- | 0/10 | 0/10 | 0/10 | 0/10 | 0/5 | 0/5 |  |  | +/- | 0/10 | 0/10 | 0/10 | 0/10 | 0/5 | 0/5 |
|  | 1+ | 2/10 | 5/10 | 2/10 | 3/10 | 3/5 | 1/5 |  |  | 1+ | 1/10 | 4/10 | 6/10 | 3/10 | 2/5 | 1/5 |
|  | 2+ | 3/10 | 1/10 | 0/10 | 1/10 | 0/5 | 0/5 |  |  | 2+ | 0/10 | 0/10 | 1/10 | 1/10 | 1/5 | 0/5 |
|  | 3+ | 0/10 | 0/10 | 0/10 | 0/10 | 0/5 | 0/5 |  |  | 3+ | 0/10 | 0/10 | 0/10 | 0/10 | 0/5 | 0/5 |
| Ketone bodies | - | 0/10 | 0/10 | 0/10 | 0/10 | 0/5 | 0/5 |  | Ketone bodies | - | 1/10 | 1/10 | 3/10 | 0/10 | 1/5 | 1/5 |
|  | +/- | 1/10 | 1/10 | 3/10 | l/10 | 0/5 | 1/5 |  |  | +/- | 9/10 | 9/10 | 7/10 | 9/10 | 3/5 | 4/5 |
|  | 1+ | 7/10 | 7/10 | 5/10 | 9/10 | 4/5 | 4/5 |  |  | 1+ | 0/10 | 0/10 | 0/10 | 1/10 | 1/5 | 0/5 |
|  | 2+ | 2/10 | 2/10 | 2/10 | 0/10 | 1/5 | 0/5 |  |  | 2+ | 0/10 | 0/10 | 0/10 | 0/10 | 0/5 | 0/5 |
|  | 3+ | 0/10 | 0/10 | 0/10 | 0/10 | 0/5 | 0/5 |  |  | 3+ | 0/10 | 0/10 | 0/10 | 0/10 | 0/5 | 0/5 |
| Specific gravity | ≤1.005 | 0/10 | 0/10 | 0/10 | 0/10 | 0/5 | 0/5 |  | Specific gravity | ≤1.005 | 0/10 | 0/10 | 0/10 | 0/10 | 0/5 | 0/5 |
|  | 1.006-1.010 | 1/10 | 1/10 | 2/10 | 0/10 | 0/5 | 0/5 |  |  | 1.006-1.010 | 0/10 | 0/10 | 0/10 | 0/10 | 0/5 | 0/5 |
|  | 1.011~1.020 | 5/10 | 4/10 | 5/10 | 9/10 | 1/5 | 3/5 |  |  | 1.011~1.020 | 3/10 | 2/10 | 2/10 | 4/10 | 1/5 | 0/5 |
|  | 1.021~1.029 | 2/10 | 3/10 | 2/10 | 1/10 | 2/5 | 1/5 |  |  | 1.021~1.029 | 3/10 | 3/10 | 3/10 | 2/10 | 0/5 | 0/5 |
|  | ≥1.030 | 2/10 | 2/10 | 1/10 | 0/10 | 2/5 | 1/5 |  |  | ≥1.030 | 4/10 | 5/10 | 5/10 | 4/10 | 4/5 | 5/5 |
| pH | ≤6.5 | 4/10 | 4/10 | 3/10 | 0/10 | 2/5 | 0/5 |  | pH | ≤6.5 | 9/10 | 8/10 | 9/10 | 5/10 | 4/5 | 5/5 |
|  | 7 | 6/10 | 6/10 | 2/10 | 4/10 | 3/5 | 5/5 |  |  | 7 | 1/10 | 2/10 | 1/10 | 3/10 | 0/5 | 0/5 |
|  | 7.5 | 0/10 | 0/10 | 5/10 | 6/10 | 0/5 | 0/5 |  |  | 7.5 | 0/10 | 0/10 | 0/10 | 2/10 | 1/5 | 0/5 |
|  | 8 | 0/10 | 0/10 | 0/10 | 0/10 | 0/5 | 0/5 |  |  | 8 | 0/10 | 0/10 | 0/10 | 0/10 | 0/5 | 0/5 |
|  | 8.5 | 0/10 | 0/10 | 0/10 | 0/10 | 0/5 | 0/5 |  |  | 8.5 | 0/10 | 0/10 | 0/10 | 0/10 | 0/5 | 0/5 |
|  | ≥9 | 0/10 | 0/10 | 0/10 | 0/10 | 0/5 | 0/5 |  |  | ≥9 | 0/10 | 0/10 | 0/10 | 0/10 | 0/5 | 0/5 |
| Protein | - | 0/10 | 0/10 | 0/10 | 0/10 | 0/5 | 0/5 |  | Protein | - | 1/10 | 0/10 | 1/10 | 0/10 | 0/5 | 0/5 |
|  | +/- | 0/10 | 0/10 | 0/10 | 0/10 | 0/5 | 0/5 |  |  | +/- | 1/10 | 2/10 | 3/10 | 2/10 | 1/5 | 0/5 |
|  | 1+ | 3/10 | 2/10 | 1/10 | 5/10 | 0/5 | 2/5 |  |  | 1+ | 7/10 | 7/10 | 5/10 | 8/10 | 1/5 | 3/5 |
|  | 2+ | 7/10 | 7/10 | 9/10 | 4/10 | 5/5 | 1/5 |  |  | 2+ | 1/10 | 1/10 | 1/10 | 0/10 | 3/5 | 2/5 |
|  | 3+ | 0/10 | 1/10 | 0/10 | 1/10 | 0/5 | 2/5 |  |  | 3+ | 0/10 | 0/10 | 0/10 | 0/10 | 0/5 | 0/5 |
| Urobilinogen | - | 10/10 | 10/10 | 10/10 | 10/10 | 5/5 | 5/5 |  | Urobilinogen | - | 10/10 | 10/10 | 10/10 | 10/10 | 5/5 | 5/5 |
|  | +/- | 0/10 | 0/10 | 0/10 | 0/10 | 0/5 | 0/5 |  |  | +/- | 0/10 | 0/10 | 0/10 | 0/10 | 0/5 | 0/5 |
|  | 1+ | 0/10 | 0/10 | 0/10 | 0/10 | 0/5 | 0/5 |  |  | 1+ | 0/10 | 0/10 | 0/10 | 0/10 | 0/5 | 0/5 |
|  | 2+ | 0/10 | 0/10 | 0/10 | 0/10 | 0/5 | 0/5 |  |  | 2+ | 0/10 | 0/10 | 0/10 | 0/10 | 0/5 | 0/5 |
|  | 3+ | 0/10 | 0/10 | 0/10 | 0/10 | 0/5 | 0/5 |  |  | 3+ | 0/10 | 0/10 | 0/10 | 0/10 | 0/5 | 0/5 |
| Occult blood | - | 9/10 | 10/10 | 9/10 | 9/10 | 5/5 | 4/5 |  | Occult blood | - | 10/10 | 10/10 | 10/10 | 10/10 | 5/5 | 5/5 |
|  | +/- | 1/10 | 0/10 | 1/10 | 1/10 | 0/5 | 1/5 |  |  | +/- | 0/10 | 0/10 | 0/10 | 0/10 | 0/5 | 0/5 |
|  | 1+ | 0/10 | 0/10 | 0/10 | 0/10 | 0/5 | 0/5 |  |  | 1+ | 0/10 | 0/10 | 0/10 | 0/10 | 0/5 | 0/5 |
|  | 2+ | 0/10 | 0/10 | 0/10 | 0/10 | 0/5 | 0/5 |  |  | 2+ | 0/10 | 0/10 | 0/10 | 0/10 | 0/5 | 0/5 |
|  | 3+ | 0/10 | 0/10 | 0/10 | 0/10 | 0/5 | 0/5 |  |  | 3+ | 0/10 | 0/10 | 0/10 | 0/10 | 0/5 | 0/5 |
|  | 4+ | 0/10 | 0/10 | 0/10 | 0/10 | 0/5 | 0/5 |  |  | 4+ | 0/10 | 0/10 | 0/10 | 0/10 | 0/5 | 0/5 |
| -: Negative; +: Positive; +/-: Trace; DK: Dark; LT: Light. n/n: Number of rats revealed each indicated observation/Number of rats per group. † C: control; S-L: Symbiota® low dose group; S-M: Symbiota® middle dose group; S-H: Symbiota® high dose group; Re-C: recovery control group; Re-H: recovery high dose group. | | | | | | | | | | | | | | | | |

| **Table S18. 90-Day Subchronic Oral Toxicity Study Organ - Urine Sediment Examination**  **Male Rats** | | | | | | | | | |  | | **Female Rats** | | | | | | | | | |
| --- | --- | --- | --- | --- | --- | --- | --- | --- | --- | --- | --- | --- | --- | --- | --- | --- | --- | --- | --- | --- | --- |
| Group† |  |  | C | S-L | S-M | S-H | Re-C | Re-H |  | | Group† | |  |  | C | S-L | S-M | S-H | Re-C | Re-H |  |
| Dose (mL/kg) |  |  | 0 | 1.5 | 5 | 15 | 0 | 15 |  | | Dose (mL/kg) | |  |  | 0 | 1.5 | 5 | 15 | 0 | 15 |  |
| Cell number (HPF) | EP | 0~1 | 10/10 | 10/10 | 10/10 | 10/10 | 5/5 | 5/5 |  | | Cell number (HPF) | | EP | 0~1 | 10/10 | 10/10 | 10/10 | 10/10 | 5/5 | 5/5 |  |
|  |  | 0~2 | 0/10 | 0/10 | 0/10 | 0/10 | 0/5 | 0/5 |  | |  |  |  | 0~2 | 0/10 | 0/10 | 0/10 | 0/10 | 0/5 | 0/5 |  |
|  |  | 1~2 | 0/10 | 0/10 | 0/10 | 0/10 | 0/5 | 0/5 |  | |  |  |  | 1~2 | 0/10 | 0/10 | 0/10 | 0/10 | 0/5 | 0/5 |  |
|  |  | 1~3 | 0/10 | 0/10 | 0/10 | 0/10 | 0/5 | 0/5 |  | |  |  |  | 1~3 | 0/10 | 0/10 | 0/10 | 0/10 | 0/5 | 0/5 |  |
|  |  | 2~4 | 0/10 | 0/10 | 0/10 | 0/10 | 0/5 | 0/5 |  | |  |  |  | 2~4 | 0/10 | 0/10 | 0/10 | 0/10 | 0/5 | 0/5 |  |
|  |  | 2~5 | 0/10 | 0/10 | 0/10 | 0/10 | 0/5 | 0/5 |  | |  |  |  | 2~5 | 0/10 | 0/10 | 0/10 | 0/10 | 0/5 | 0/5 |  |
|  |  | 3~5 | 0/10 | 0/10 | 0/10 | 0/10 | 0/5 | 0/5 |  | |  |  |  | 3~5 | 0/10 | 0/10 | 0/10 | 0/10 | 0/5 | 0/5 |  |
|  | WBC | 0~1 | 10/10 | 9/10 | 8/10 | 10/10 | 5/5 | 3/5 |  | |  |  | WBC | 0~1 | 10/10 | 9/10 | 8/10 | 10/10 | 5/5 | 5/5 |  |
|  |  | 0~2 | 0/10 | 1/10 | 2/10 | 0/10 | 0/5 | 1/5 |  | |  |  |  | 0~2 | 0/10 | 1/10 | 2/10 | 0/10 | 0/5 | 0/5 |  |
|  |  | 0~3 | 0/10 | 0/10 | 0/10 | 0/10 | 0/5 | 0/5 |  | |  |  |  | 0~3 | 0/10 | 0/10 | 0/10 | 0/10 | 0/5 | 0/5 |  |
|  |  | 1~2 | 0/10 | 0/10 | 0/10 | 0/10 | 0/5 | 0/5 |  | |  |  |  | 1~2 | 0/10 | 0/10 | 0/10 | 0/10 | 0/5 | 0/5 |  |
|  |  | 1~3 | 0/10 | 0/10 | 0/10 | 0/10 | 0/5 | 0/5 |  | |  |  |  | 1~3 | 0/10 | 0/10 | 0/10 | 0/10 | 0/5 | 0/5 |  |
|  |  | 2~3 | 0/10 | 0/10 | 0/10 | 0/10 | 0/5 | 0/5 |  | |  |  |  | 2~3 | 0/10 | 0/10 | 0/10 | 0/10 | 0/5 | 0/5 |  |
|  |  | 2~5 | 0/10 | 0/10 | 0/10 | 0/10 | 0/5 | 1/5 |  | |  |  |  | 2~5 | 0/10 | 0/10 | 0/10 | 0/10 | 0/5 | 0/5 |  |
|  |  | 3~4 | 0/10 | 0/10 | 0/10 | 0/10 | 0/5 | 0/5 |  | |  |  |  | 3~4 | 0/10 | 0/10 | 0/10 | 0/10 | 0/5 | 0/5 |  |
|  |  | 3~5 | 0/10 | 0/10 | 0/10 | 0/10 | 0/5 | 0/5 |  | |  |  |  | 3~5 | 0/10 | 0/10 | 0/10 | 0/10 | 0/5 | 0/5 |  |
|  |  | 4~5 | 0/10 | 0/10 | 0/10 | 0/10 | 0/5 | 0/5 |  | |  |  |  | 4~5 | 0/10 | 0/10 | 0/10 | 0/10 | 0/5 | 0/5 |  |
|  |  | 4~6 | 0/10 | 0/10 | 0/10 | 0/10 | 0/5 | 0/5 |  | |  |  |  | 4~6 | 0/10 | 0/10 | 0/10 | 0/10 | 0/5 | 0/5 |  |
|  |  | >6 | 0/10 | 0/10 | 0/10 | 0/10 | 0/5 | 0/5 |  | |  |  |  | >6 | 0/10 | 0/10 | 0/10 | 0/10 | 0/5 | 0/5 |  |
|  | RBC | 0~1 | 10/10 | 10/10 | 10/10 | 10/10 | 5/5 | 3/5 |  | |  |  | RBC | 0~1 | 10/10 | 10/10 | 10/10 | 10/10 | 5/5 | 5/5 |  |
|  |  | 0~2 | 0/10 | 0/10 | 0/10 | 0/10 | 0/5 | 2/5 |  | |  |  |  | 0~2 | 0/10 | 0/10 | 0/10 | 0/10 | 0/5 | 0/5 |  |
|  |  | 0~3 | 0/10 | 0/10 | 0/10 | 0/10 | 0/5 | 0/5 |  | |  |  |  | 0~3 | 0/10 | 0/10 | 0/10 | 0/10 | 0/5 | 0/5 |  |
|  |  | 0~4 | 0/10 | 0/10 | 0/10 | 0/10 | 0/5 | 0/5 |  | |  |  |  | 0~4 | 0/10 | 0/10 | 0/10 | 0/10 | 0/5 | 0/5 |  |
|  |  | 1~2 | 0/10 | 0/10 | 0/10 | 0/10 | 0/5 | 0/5 |  | |  |  |  | 1~2 | 0/10 | 0/10 | 0/10 | 0/10 | 0/5 | 0/5 |  |
|  |  | 1~3 | 0/10 | 0/10 | 0/10 | 0/10 | 0/5 | 0/5 |  | |  |  |  | 1~3 | 0/10 | 0/10 | 0/10 | 0/10 | 0/5 | 0/5 |  |
|  |  | 2~3 | 0/10 | 0/10 | 0/10 | 0/10 | 0/5 | 0/5 |  | |  |  |  | 2~3 | 0/10 | 0/10 | 0/10 | 0/10 | 0/5 | 0/5 |  |
|  |  | 2~4 | 0/10 | 0/10 | 0/10 | 0/10 | 0/5 | 0/5 |  | |  |  |  | 2~4 | 0/10 | 0/10 | 0/10 | 0/10 | 0/5 | 0/5 |  |
|  |  | 3~5 | 0/10 | 0/10 | 0/10 | 0/10 | 0/5 | 0/5 |  | |  |  |  | 3~5 | 0/10 | 0/10 | 0/10 | 0/10 | 0/5 | 0/5 |  |
|  |  | 4~6 | 0/10 | 0/10 | 0/10 | 0/10 | 0/5 | 0/5 |  | |  |  |  | 4~6 | 0/10 | 0/10 | 0/10 | 0/10 | 0/5 | 0/5 |  |
|  |  | >6 | 0/10 | 0/10 | 0/10 | 0/10 | 0/5 | 0/5 |  | |  |  |  | >6 | 0/10 | 0/10 | 0/10 | 0/10 | 0/5 | 0/5 |  |
| Crystals (LPF) | None | | 4/10 | 5/10 | 6/10 | 7/10 | 2/5 | 4/5 |  | | Crystals (LPF) | | None | | 6/10 | 6/10 | 6/10 | 6/10 | 4/5 | 5/5 |  |
|  | Triple phosphate | | 6/10 | 5/10 | 4/10 | 3/10 | 3/5 | 1/5 |  | |  |  | Triple phosphate | | 4/10 | 4/10 | 4/10 | 4/10 | 1/5 | 0/5 |  |
| EP: Epithelial cell; WBC: White blood cell; RBC: Red blood cell; HPF: High power field; LPF: Low power field.  n/n: Number of rats revealed each indicated observation/Number of rats per group. †C: control; S-L: Symbiota® low dose group; S-M: Symbiota® middle dose group; S-H: Symbiota® high dose group; Re-C: recovery control group; Re-H: recovery high dose group. | | | | | | | | | | | | | | | | | | | | | |

| **Table S19. 90-Day Subchronic Oral Toxicity Study Organ - Incidence of Ophthalmologic Abnormalities** | | | | | | | | | |
| --- | --- | --- | --- | --- | --- | --- | --- | --- | --- |
|  | | | | | | | | | |
| **Main study** | | | | | | | | | |
|  | Prior to administration | | | | Before scheduled sacrifice | | | | |
| Group† | C | | S-H | | C | | S-H | | |
| Dose (mL/kg) | 0 | | 15 | | 0 | | 15 | | |
| Sex | Male | Female | Male | Female | Male | Female | Male | Female |  |
| Abnormality | 0/10 | 0/10 | 0/10 | 0/10 | 0/10 | 0/10 | 0/10 | 0/10 |  |
|  |  |  |  |  |  |  |  |  |  |
| **Recovery study** | | | | | | | | | |
|  | Prior to administration | | | | Before scheduled sacrifice | | | | |
| Group† | C | | Re-H | | C | | Re-H | | |
| Dose (mL/kg) | 0 | | 15 | | 0 | | 15 | | |
| Sex | Male | Female | Male | Female | Male | Female | Male | Female |  |
| Abnormality | 0/5 | 0/5 | 0/5 | 0/5 | 0/5 | 0/5 | 0/5 | 0/5 |  |
| n/n: Number of rats with abnormalities/Number of rats in group. †C: control; S-H: Symbiota® high dose group; Re-C: recovery control group; Re-H: recovery high dose group. | | | | | | | | | |

| **Table S20. 90-Day Subchronic Oral Toxicity Study Organ - Incidence of Histopathological Lesions**  **Main Study** | | | | | |
| --- | --- | --- | --- | --- | --- |
| Organ | Lesions† | Group | | | |
|  |  | Control | | S-H^#^ | |
|  |  | Male | Female | Male | Female |
| Adrenal gland |  | - | - | - | - |
| Aorta |  | - | - | - | - |
| Brain |  |  |  |  |  |
| Fore |  | - | - | - | - |
| Middle |  | - | - | - | - |
| Cerebellum |  | - | - | - | - |
| Bone |  | - | - | - | - |
| Bone marrow |  | - | - | - | - |
| Cervix |  | N | - | N | - |
| Coagulating gland |  |  |  |  |  |
|  | Infiltration, mononuclear cell, interstitial, multifocal, minimal to slight | 5/10 | N | 7/10 | N |
| Epididymis |  | - | N | - | N |
| Esophagus |  | - | - | - | - |
| Eyes |  | - | - | - | - |
| Harderian gland |  |  |  |  |  |
|  | Infiltration, mononuclear cell, interstitial, multifocal, minimal to slight | 2/10 | 3/10 | 2/10 | 2/10 |
| Heart |  |  |  |  |  |
|  | Infiltration, mononuclear cell, focal, minimal to slight | 1/10 | - | 1/10 | 1/10 |
| Intestine, small |  |  |  |  |  |
| Duodenum |  | - | - | - | - |
| Jejunum |  | - | - | - | - |
| Ileum |  | - | - | - | - |
| Intestine, large |  |  |  |  |  |
| Cecum |  | - | - | - | - |
| Colon |  | - | - | - | - |
| Rectum |  | - | - | - | - |
| Kidney |  |  |  |  |  |
|  | Cast, tubule, multifocal, minimal to slight | - | 1/10 | 1/10 | - |
|  | Infarct, tubule, focal, slight | - | - | 1/10 | - |
|  | Infiltration, mononuclear cell, interstitial, focal, minimal to slight | 2/10 | 4/10 | - | 1/10 |
|  | Mineralization, tubule, multifocal, minimal to moderate | 1/10 | 8/10 | 4/10 | 8/10 |
| Liver |  | - | - | - | - |
| Lung |  |  |  |  |  |
|  | Aggregation, macrophage, focal, slight | 1/10 | - | - | - |
| Lymph node |  |  |  |  |  |
| Cervival |  | - | - | - | - |
| Mesenteric |  | - | - | - | - |
| Mammary gland |  | - | - | - | - |
| Optic nerve |  | - | - | - | - |
| Ovary |  | N | - | N | - |
| Oviduct |  | N | - | N | - |
| Pancreas |  | - | - | - | - |
| Parathyroid gland |  | - | - | - | - |
| Pituitary |  | - | - | - | - |
| Prostate gland |  | - | N | - | N |
| Salivary gland |  |  |  |  |  |
| Mandibular lobe |  | - | - | - | - |
| Sublingual lobe |  | - | - | - | - |
| Sciatic nerve |  | - | - | - | - |
| Seminal vesicle |  | - | N | - | N |
| Skeletal muscle |  | - | - | - | - |
| Skin |  | - | - | - | - |
| Spinal cord |  |  |  |  |  |
| Cervical |  | - | - | - | - |
| Lumbar |  | - | - | - | - |
| Thoracic |  | - | - | - | - |
| Spleen |  | - | - | - | - |
| Stomach |  |  |  |  |  |
|  | Hemorrhage, submucosa, focal, slight | - | - | 1/10 | - |
|  | Inflammation, submucosa, focal, slight | - | - | 1/10 | - |
| Testes |  | - | N | - | N |
| Thymus |  | - | - | - | - |
| Thyroid gland |  | - | - | - | - |
| Tongue |  | - | - | - | - |
| Trachea |  | - | - | - | - |
| Urinary bladder |  | - | - | - | - |
| Uterus |  | N | - | N | - |
| Vagina |  | N | - | N | - |
| Incidence: Affected rats/ Total examined rats (n = 10) -: No significant lesions; N: No tissue available. †Degree of lesions was graded from one to five depending on severity: 1 = minimal (≤1%);2=slight (1-25%)；3= moderate (26-50%); 4 = moderate/severe (51-75%); 5 = severe/high (76-100%). ^#^S-H: Symbiota® high dose group | | | | | |
|  |  |  |  |  |  |
| **Recovery Study** | | | | | |
| Organ | Lesions† | Group^#^ | | | |
|  |  | Re-C | | Re-H | |
|  |  | Male | Female | Male | Female |
| Adrenal gland |  | - | - | - | - |
| Aorta |  | - | - | - | - |
| Brain |  |  |  |  |  |
| Fore |  | - | - | - | - |
| Middle |  | - | - | - | - |
| Cerebellum |  | - | - | - | - |
| Bone |  | - | - | - | - |
| Bone marrow |  | - | - | - | - |
| Cervix |  | N | - | N | - |
| Coagulating gland |  |  |  |  |  |
|  | Infiltration, mononuclear cell, interstitial, multifocal, minimal to slight | 3/5 | N | 4/5 | N |
| Epididymis |  | - | N | - | N |
| Esophagus |  | - | - | - | - |
| Eyes |  | - | - | - | - |
| Harderian gland |  |  |  |  |  |
|  | Infiltration, mononuclear cell, interstitial, multifocal, minimal to slight | - | - | 2/5 | 1/5 |
| Heart |  |  |  |  |  |
|  | Infiltration, mononuclear cell, focal, minimal to slight | 1/5 | - | - | 2/5 |
| Intestine, small |  |  |  |  |  |
| Duodenum |  | - | - | - | - |
| Jejunum |  | - | - | - | - |
| Ileum |  | - | - | - | - |
| Intestine, large |  |  |  |  |  |
| Cecum |  | - | - | - | - |
| Colon |  | - | - | - | - |
| Rectum |  | - | - | - | - |
| Kidney |  |  |  |  |  |
|  | Calculus, pelvis, focal, slight | 1/5 | - | - | - |
|  | Cast, tubule, multifocal, slight to moderate | - | - | 2/5 | - |
|  | Infarct, tubule, focal, slight | - | 1/5 | - | - |
|  | Infiltration, mononuclear cell, interstitial, focal, minimal | 1/5 | 1/5 | 3/5 | 1/5 |
|  | Mineralization, tubule, multifocal, minimal to moderate | - | 4/5 | 2/5 | 3/5 |
| Liver |  | - | - | - | - |
| Lung |  |  |  |  |  |
|  | Aggregation, macrophage, focal, slight | - | - | 1/5 | 1/5 |
| Lymph node |  |  |  |  |  |
| Cervival |  | - | - | - | - |
| Mesenteric |  | - | - | - | - |
| Mammary gland |  | - | - | - | - |
| Optic nerve |  | - | - | - | - |
| Ovary |  | N | - | N | - |
| Oviduct |  | N | - | N | - |
| Pancreas |  | - | - | - | - |
| Parathyroid gland |  | - | - | - | - |
| Pituitary |  | - | - | - | - |
| Prostate gland |  | - | N | - | N |
| Salivary gland |  |  |  |  |  |
| Mandibular lobe |  | - | - | - | - |
| Sublingual lobe |  | - | - | - | - |
| Sciatic nerve |  | - | - | - | - |
| Seminal vesicle |  | - | N | - | N |
| Skeletal muscle |  | - | - | - | - |
| Skin |  | - | - | - | - |
| Spinal cord |  |  |  |  |  |
| Cervical |  | - | - | - | - |
| Lumbar |  | - | - | - | - |
| Thoracic |  | - | - | - | - |
| Spleen |  | - | - | - | - |
| Stomach |  | - | - | - | - |
| Testes |  | - | N | - | N |
| Thymus |  | - | - | - | - |
| Thyroid gland |  | - | - | - | - |
| Tongue |  | - | - | - | - |
| Trachea |  | - | - | - | - |
| Urinary bladder |  | - | - | - | - |
| Uterus |  | N | - | N | - |
| Vagina |  | N | - | N | - |
| Incidence: Affected rats/ Total examined rats (n = 5) -: No significant lesions; N: No tissue available. †Degree of lesions was graded from one to five depending on severity: 1 = minimal (≤1%);2=slight (1-25%)；3= moderate (26-50%); 4 = moderate/severe (51-75%); 5 = severe/high (76-100%). ^#^Re-C: recovery control group; Re-H: recovery high dose group. | | | | | |

| **Table S21. 90-Day Subchronic Oral Toxicity Study Organ - Serum Thyroid Hormones** | | | | | | | | |
| --- | --- | --- | --- | --- | --- | --- | --- | --- |
| **Main study** | | | | | | | | |
| Group† | C | | S-L | | S-M | | S-H | |
| Dose (mL/kg) | 0 | | 1.5 | | 5 | | 15 | |
| Sex | Male | Female | Male | Female | Male | Female | Male | Female |
| Test parameters | | | | | | | | |
| T3 (ng/mL) | 1.2561 ± 0.1135 | 0.4891 ± 0.1288 | 1.4143 ± 0.2058 | 0.4829 ± 0.0514 | 1.3300 ± 0.2801 | 0.4854 ± 0.0803 | 1.2530 ± 0.1414 | 0.4904 ± 0.1159 |
| T4 (ng/mL) | 56.7334 ± 8.1005 | 57.0374 ± 7.7700 | 56.1104 ± 6.7124 | 56.4900 ± 8.5199 | 57.9285 ± 9.7796 | 57.6125 ± 5.4650 | 56.6265 ± 6.4310 | 58.6981 ± 5.7782 |
| TSH (ng/mL) | 1.1722 ± 0.4260 | 1.3171 ± 0.3767 | 1.1357 ± 0.4808 | 1.2923 ± 0.2989 | 1.1913 ± 0.4725 | 1.3500 ± 0.2493 | 1.2871 ± 0.4520 | 1.3086 ± 0.4864 |
|  |  |  |  |  |  |  |  |  |
| **Recovery study** | | | | | | | | |
| Group† | Re-C | | | | Re-H | | | |
| Dose (mL/kg) | 0 | | | | 15 | | | |
| Sex | Male | | Female | | Male | | Female | |
| Test parameters | | | | | | | | |
| T3 (ng/mL) | 1.1810 ± 0.1066 | | 0.4880 ± 0.1587 | | 1.1876 ± 0.1856 | | 0.4960 ± 0.1641 | |
| T4 (ng/mL) | 58.7960 ± 6.9614 | | 57.8992 ± 6.4414 | | 52.7438 ± 7.7569 | | 58.6000 ± 5.1988 | |
| TSH (ng/mL) | 1.2100 ± 0.5271 | | 1.2554 ± 0.3708 | | 1.1806 ± 0.3203 | | 1.3090 ± 0.3541 | |
| Data were presented as mean ± S.D. of 10 animals per group in main study; 5 animals per group in recovery study. †C: control; S-L: Symbiota® low dose group; S-M: Symbiota® middle dose group; S-H: Symbiota® high dose group; Re-C: recovery control group; Re-H: recovery high dose group. | | | | | | | | |
|  | | | | | | | | |
